# Effect of Repeated Heat–Moisture Treatment Temperature on the Multi-Scale Structure, Physicochemical Properties, Rheological Behavior, and In Vitro Digestibility of Hard Proso Millet Starch

**DOI:** 10.3390/foods15132308

**Published:** 2026-06-29

**Authors:** Meiqi Dong, Daiyan Chao, Yajing Cao, Xingyu Guo, Chengmei Liu, Jianguo Xu, Yan Ding, Yonghua Wei, Xiaojiang Wu

**Affiliations:** 1 College of Food Science, Shanxi Normal University, Taiyuan 030000, China; dmq920413@163.com (M.D.); 17639923214@163.com (D.C.); 15135399777@163.com (Y.C.); xjg71@163.com (J.X.); weiyonghua1983@126.com (Y.W.); 2 College of Food Science and Engineering, Ocean University of China, Qingdao 266404, China; gxy245403757@163.com; 3 State Key Laboratory of Food Science and Technology, College of Food Science and Technology, Nanchang University, Nanchang 330047, China; liuchengmei@ncu.edu.cn; 4 Department of Life Science, Linfen Vocational and Technial College, Linfen 041000, China; dingyanborre@163.com

**Keywords:** hard proso millet starch, repeated heat–moisture treatment, in vitro digestibility

## Abstract

Repeated heat–moisture treatment (RHMT) is an efficient approach for modifying starch. However, the role of treatment temperature, a critical parameter, remains poorly understood. Therefore, this study investigated the effects of RHMT temperatures (80, 100, 120 °C) and cycles (1, 3, 5, 7) on the multi-scale structure and in vitro digestibility of hard proso millet starch, using native starch as a control. Compared with the severe 120 °C treatment, processing at 100 °C better preserved double-helical organization (supported by moderately retained enthalpy, Δ*H*) and short-range order, while maintaining granule integrity. These structural retentions restricted swelling, improved pasting stability, and reinforced the macroscopic gel network. Furthermore, multivariate analysis suggested that the rigidified internal granular architecture delayed initial enzymatic hydrolysis, maximizing slowly digestible starch (SDS) formation (47.44% in 100-RHMT-5). Conversely, 120 °C caused severe granular collapse and a drastic drop in Δ*H*, diminishing gel elasticity and triggering a surge in rapidly digestible starch (RDS, 59%). Overall, 100 °C RHMT yields an SDS-enriched starch, which may be a promising ingredient for the development of starch-based foods with slower in vitro digestibility.

## 1. Introduction

Proso millet (*Panicum miliaceum* L.) is a climate-resilient cereal widely grown in arid regions due to its short growth cycle and tolerance to drought and heat [1]. It is also a nutritious food source, rich in essential amino acids, minerals, and polyphenols with health benefits such as antioxidant and lipid-lowering effects [2,3]. Based on amylose content, millet is classified into soft and hard types. Hard millet, rich in amylose, is a promising ingredient for functional foods. Starch, comprising 58.1–77.9% of the grain, governs its processing functionality and nutritional profile [3]. However, native hard millet starch often suffers from poor processing stability (high retrogradation, low shear resistance). Moreover, its inherently high resistant starch (RS) content is easily destroyed during conventional thermal processing, leading to rapid digestion in final food products [4]. Therefore, modification is required to regulate the multi-scale structure of starch, improve its physicochemical properties, and modulate its digestibility.

Heat–moisture treatment (HMT) is a sustainable, clean-label physical modification technique for modulating starch functionality [5,6]. The process involves heating starch at restricted moisture (<35%) and high temperatures (80–140 °C). HMT typically increases pasting temperature and thermal stability but reduces granule swelling, peak viscosity, and retrogradation. It can also promote the formation of resistant starch (RS) and slowly digestible starch (SDS). These modification effects are strongly dependent on processing parameters (moisture, temperature, duration) as well as the starch’s botanical origin and intrinsic structure [4].

Compared with conventional HMT, repeated heat–moisture treatment (RHMT) facilitates iterative molecular and structural reorganization, enhancing starch structural stability, decreasing enzymatic digestibility, and creating more controllable physicochemical properties. For instance, RHMT has been shown to improve structural perfection and thermal stability of waxy maize starch by enhancing molecular order and inducing A-type crystalline transition [7], and to increase SDS content and gelatinization temperature of sweet potato starch while reducing granule swelling [8]. As an effective physical modification method, RHMT is influenced by processing parameters including moisture content, duration, and temperature. Temperature is particularly crucial, as it governs molecular chain mobility in amorphous regions and alters crystalline structures. These structural changes directly impact the viscoelastic, rheological, and enzymatic hydrolysis properties of starch. However, most existing studies have focused on the number of RHMT cycles, while systematic investigations into the effects of different treatment temperatures are scarce.

Given the critical role of temperature in governing molecular chain mobility, we hypothesized that the structural reorganization and digestion resistance of hard proso millet starch are uniquely regulated by a thermal threshold during RHMT, where moderate heat facilitates structural perfection while excessive heat induces crystalline disruption. Therefore, this study systematically investigated the effects of RHMT at various temperatures (80, 100, and 120 °C) and cycles (1, 3, 5, and 7) on the multi-scale structure, pasting and rheological properties, and in vitro digestibility of hard proso millet starch. The findings aim to elucidate the relationships between starch structure, its functional properties, and digestibility, as governed by temperature, providing a mechanistic basis for the development of starch-based foods with slower in vitro digestibility.

## 2. Materials and Methods

### 2.1. Materials

Hard proso millet was sourced from Kelan County, Shanxi Province. Sigma-Aldrich (St. Louis, MO, USA) supplied both porcine pancreatic α-amylase (50 U/mg) and amyloglucosidase (70 U/mg). For glucose analysis, a GOPOD kit from Megazyme Ltd. (Wicklow, Ireland) was employed. All chemical reagents employed were of analytical grade, and distilled water served as the solvent for all experimental procedures.

### 2.2. Starch Isolation

Milled hard proso millet (100-mesh, 150 μm) was mixed with 0.3% NaOH solution at a 1:10 (*w*/*v*) ratio, stirred magnetically at 30 °C for 15 min, and then held at 25 °C for 24 h. The mixture was subsequently filtered through a 200-mesh sieve (75 μm) and centrifuged at 4000× *g* for 15 min to obtain a precipitate. The solid residue was washed with distilled water until the filtrate was clear, neutralized to pH 7.0 with 1 M HCl, and dried at 40 °C. The dried sample was finally ground to obtain native starch (NS). The chemical composition and particle characteristics of the obtained NS were as follows: amylose content 24.7 ± 1.2%, moisture content 11.3 ± 0.5%, residual protein 0.32 ± 0.05%, lipid 0.18 ± 0.03%, ash 0.21 ± 0.02%, and starch purity 98.7 ± 0.3%. The granules were polygonal or spherical, with particle sizes ranging from 5.9 to 10.2 μm and an average size of 6.82 ± 0.4 μm.

### 2.3. Repeated Heat–Moisture Treatment

Hard proso millet starch (30 g, dry basis) was adjusted to 30% moisture content by adding calculated amounts of distilled water. The mixture was placed in 100 mL tightly sealed glass jars and equilibrated at 4 °C for 24 h to ensure uniform moisture distribution [9]. For RHMT, the hermetically sealed jars were heated in a forced-air oven at 80, 100, or 120 °C for 2 h. One RHMT cycle consisted of 2 h of heating followed by cooling to room temperature. Crucially, the jars remained sealed throughout the repeated cycles (1, 3, 5, or 7) to prevent moisture evaporation, and the moisture content was verified by weighing the jars before and after the total treatment, with no significant loss (<0.5%) observed across all temperatures. After the final cycle, the treated samples were dried at 40 °C for 24 h, milled, sieved through a 100-mesh screen, and stored in airtight bags. Samples were labeled as T-RHMT-C, where T denotes temperature and C denotes the cycle number (e.g., 80-RHMT-1).

### 2.4. Determination of Pasting Properties

To determine the pasting behavior, a Rapid Visco Analyzer (RVA; Perten Instruments, Hägersten, Sweden) was utilized. Starch suspensions containing 3.0 g of dry starch and 25 mL of distilled water were subjected to a standard heating–cooling program. The starch slurry was subjected to the following temperature profile: an initial hold at 50 °C for 1 min, heating to 95 °C over 3.7 min, a plateau at 95 °C for 2.5 min, cooling to 50 °C over 3.8 min, and a final hold at 50 °C for 2 min. The paddle speed was set to 960 rpm for the first 10 s, then reduced to 160 rpm for the remainder of the test.

### 2.5. Determination of Rheological Properties

A 12% (*w*/*v*) starch slurry (3 g starch in 25 mL water) was prepared and fully gelatinized. The obtained starch gel was cooled to ambient temperature and equilibrated for 30 min, then evenly loaded onto the 50-mm parallel plate of an MCR 302 rheometer (Anton Paar, Graz, Austria) with a measurement gap of 1 mm. Steady-state flow and dynamic oscillatory tests were subsequently performed to evaluate the flow and viscoelastic properties. All measurements were carried out in triplicate using independently prepared samples.

#### 2.5.1. Dynamic Rheological Properties

The frequency sweep was performed at a fixed strain of 1% (within LVR) over the frequency range of 0.1–20 Hz. Storage modulus (*G′*), loss modulus (*G″*) and loss tangent (tan *δ*) were determined throughout the test.

#### 2.5.2. Static Rheological Properties

##### Determination of Apparent Viscosity

The apparent viscosity (*η*) of starch paste was measured at 25 °C over shear rates ranging from 0.1 to 300 s^−1^. Experimental data were fitted to the Power-Law model, as shown in Equation (1):

*τ* = *Kγ^n^* (1)

where *τ* is shear stress (Pa), *K* is the consistency coefficient (Pa·s^n^), *γ* is shear rate (s^−1^), and *n* refers to the flow behavior index.

##### Creep and Recovery

Creep–recovery measurements were carried out at 25 °C. Samples were subjected to 300 s creep at 8.15 Pa and 5 min recovery at 0 Pa. Shear strain versus time was continuously monitored. Creep compliance (*J_C_*, Pa^−1^) was defined as shear strain divided by applied stress. The four-parameter Burgers model (Equation (2)) was used for data fitting:

(2) $$J_{C}\;=\;J_{0C}+\;J_{1C}\;[1\;-\;\exp\;(-\;\frac{t}{\lambda_{1C}})]\;+\;\frac{t}{\mu_{0}}$$

where *J*_0*C*_ is the instantaneous elastic compliance (Pa^−1^) of the Maxwell unit, *J*_1*C*_ is the delayed elastic compliance (Pa^−1^) of the Kelvin–Voigt unit, *λ*_1*C*_ is the retardation time (s) of the Kelvin–Voigt component, *μ*_0_ denotes the Newtonian viscosity (Pa·s) of the Maxwell dashpot, and *t* is the testing time (s).

### 2.6. In Vitro Digestibility

#### 2.6.1. In Vitro Digestion Experiment

In vitro starch digestibility was determined according to our previously reported method [10], occurring without pre-gelatinization prior to enzymatic hydrolysis. Specifically, starch samples (200 mg, dry basis) were dispersed in 10.0 mL of sodium acetate buffer (0.2 M, pH 5.2, containing 1.0 mM CaCl_2_). The suspension was equilibrated at 37 °C for 10 min with constant magnetic stirring at 180 rpm. Subsequently, 1.5 mL of an enzyme mixture, containing porcine pancreatic α-amylase (100 U/mL) and amyloglucosidase (100 U/mL), was added to initiate hydrolysis. Aliquots (500 μL) were withdrawn at specific time intervals (0, 10, 20, 30, 60, 90, 120, 150, and 180 min) and immediately mixed with 4.0 mL of absolute ethanol to inactivate the enzymes, followed by centrifugation at 3500× *g* for 10 min. The glucose concentration in the supernatant was determined using the GOPOD assay kit (Megazyme, Wicklow, Ireland). Free glucose (*FG*) in the starch samples was measured using a control group without enzyme addition. The contents of the starch fractions, namely RDS, SDS, and RS, were ascertained in accordance with the stoichiometric equations described by Englyst et al. [11]:

(3) $$\mathrm{RDS}\left(\%\right)=\frac{G_{20}-F_{G}}{TS}\times 0.9\times 100$$

(4) $$\mathrm{SDS}\left(\%\right)=\frac{G_{120}-G_{20}}{TS}\times 0.9\times 100$$

RS(%) = 100 − RDS(%) − SDS(%)(5)

where *G*_20_ and *G*_120_ represent the glucose weight released after 20 and 120 min of hydrolysis, respectively; *FG* is the free glucose content; *TS* is the total weight of the starch sample; and 0.9 is the conversion factor from glucose to starch.

#### 2.6.2. Logarithm of Slope (LOS) Plot Analysis and Fitting of the Combined Parallel-Sequential (CPS) Digestion Kinetics Model

The fitting procedure herein referred to the method established by Yang et al. [12]. Under normal conditions, starch digestion curves conform to the first-order kinetic equation, which can be described by the following equation:

*C_t_* = *C*_0_ + (*C_∞_* − *C*_0_) × (1 − e^−*kt*^) (6)

where *C_t_* and *C*_0_ are the digestion rates at time t and 0; *C_∞_* is the maximum digestion percentage at the end of the reaction; and *k* is the rate constant.

The logarithm of slope (LOS) plot method was applied to the digestion curves to evaluate the existence of multiple digestion phases.

(7) $$\ln\frac{dC(t)}{dt}=\;\ln(C_{\infty }\;-\;C_{0})\;-\;kt$$

It was speculated that the digestive system comprised two separate digestible components, both adhering to pseudo-first-order kinetics. Therefore, the total starch digestion extent at time t can be described by the following equation:

*C*(*t*) = *C*_0_ + (*C*_1∞_) × (1 − e^−*k*1*t*^) + If (*t* ≥ *t*_2*start*_, ((*C*_2∞_) × (1 − e^−*k*2(*t*−*t*2*start*)^)), 0) (8)

*C*_1*∞*_ and *C*_2*∞*_ denote the maximum digestion percentages for Fraction 1 and Fraction 2, respectively, while *k*_1_ and *k*_2_ are their corresponding pseudo-first-order digestion rate constants. The parameter *t*_2*start*_ denotes the onset time for the digestion of the second starch fraction [13].

### 2.7. Differential Scanning Calorimetry (DSC)

A differential scanning calorimeter (DSC 3, Mettler Toledo, Greifensee, Switzerland) was used to characterize the thermal behaviors of the samples [10]. Approximately 3.0 mg of dry sample was placed into an aluminum pan, and distilled water was supplemented at a sample-to-water ratio of 1:3.5 with a microsyringe. The sealed pan was kept at room temperature for 24 h to reach moisture equilibrium. An empty aluminum pan was applied as the reference. The heating scan was performed from 30 °C to 120 °C at a heating rate of 10 °C/min. The characteristic thermal transition parameters, including onset temperature (*To*), peak temperature (*Tp*), conclusion temperature (*Te*), and gelatinization enthalpy (Δ*H*), were calculated using the STARe thermal analysis software 16.30 (Mettler Toledo, Greifensee, Switzerland).

### 2.8. Long-Range Structure

XRD measurements were performed using an X-ray diffractometer equipped with Cu-K*α* radiation [14,15], operating at a voltage of 40 kV and a current of 100 mA. The diffractometer was set to scan over a 2*θ* range of 5° to 40° at a scanning rate of 5°/min with a step size of 0.02°. The relative crystallinity (*RC*) was calculated using Origin 8.5 software according to the following equation:

(9) $$\mathrm{RC}(\%)=\frac{\mathrm{Ac}}{\mathrm{Ac}+\mathrm{Aa}}\times 100$$

where *Ac* and *Aa* represent the areas of the crystalline region and the amorphous region in the X-ray diffraction pattern, respectively.

### 2.9. Short-Range Structure

Fourier transform infrared (FTIR) spectra were recorded in the 4000–800 cm^−1^ range at 4 cm^−1^ resolution with 64 scans [16]. The FTIR spectra in the range of 1200–800 cm^−1^ were subjected to automatic baseline correction and deconvolution in absorbance mode using Omnic 8.2 software (Thermo Fisher Scientific Inc., Waltham, MA, USA). A Lorentzian line shape was assumed for deconvolution, with a half-height width (HHW) of 19 cm^−1^ and a resolution enhancement factor of 1.9. The calibrated peak heights at 995 cm^−1^, 1022 cm^−1^, and 1047 cm^−1^ were recorded to calculate the absorbance height ratios of 1047/1022 and 995/1022.

### 2.10. Scanning Electron Microscope (SEM)

SEM was employed to observe the samples. Specimens were affixed to stubs and metallized with gold; images were captured at 10.0 kV and 3000× magnification.

### 2.11. Statistical Analysis Methods

All experiments were conducted in independent triplicate, and results are presented as mean ± SD (*n* = 3). Factorial data were analyzed via two-way ANOVA with treatment temperature (*T*) and number of cycles (*C*) as fixed factors. Significant *T* × *C* interactions were resolved using simple main effects via estimated marginal means (EMMEANS) with Bonferroni adjustment (*p* < 0.05). Without significant interaction, main effects were separated using Duncan’s multiple range test (*p* < 0.05). The independent control (NS) was excluded from the two-way ANOVA, and comparisons between treated samples and NS were performed using Dunnett’s two-sided test (*p* < 0.05). These analyses were executed using SPSS 22.0 (IBM Corp., Armonk, NY, USA). Furthermore, to elucidate multivariate relationships among structural, rheological, thermal, and digestive parameters, hierarchical clustering, Pearson correlation, and Principal Component Analysis (PCA) were conducted using Origin 2022 (OriginLab Corp., Northampton, MA, USA).

## 3. Results and Discussion

### 3.1. Pasting Properties

The effects of RHMT at different temperatures on the pasting properties of hard proso millet starch are shown in Table 1, and the corresponding two-way ANOVA results are summarized in Table 2. Temperature (*T*), number of cycles (*C*), and their interaction (*T* × *C*) significantly affected the pasting properties of samples (Table 2), indicating that the impact of temperature depended on the cycle number and vice versa.

Compared with NS, all RHMT-treated samples exhibited significantly higher pasting temperature (PT) values (*p* < 0.05; Table 1), indicating enhanced thermal resistance of starch granules after treatment. The highest PT was observed in 100-RHMT-7 (86.13 °C). However, the increase in PT was not strictly proportional to treatment temperature, as PT values at 120 °C were not consistently higher than those at 100 °C, suggesting that 100 °C may be more favorable for structural rearrangement, whereas 120 °C may induce slight granular disruption or partial pre-gelatinization. This increase in PT is likely associated with strengthened molecular interactions and enhanced crystalline order within starch granules, which require more energy to initiate swelling [17,18,19]. In contrast, RHMT reduced the peak viscosity (PV) in a *T* × *C*-dependent manner (Table 1 and Table 2). Relative to NS, PV decreases were significant for all 100 °C treatments and for 120-RHMT-1/3/7, whereas PV changes at 80 °C were not always significant (only 80-RHMT-5 showed a significant decrease, Table 1). The lowest PV was found in 120-RHMT-7 (3552 cP), which was significantly lower than that of NS (*p* < 0.05), indicating restricted granule swelling. Accordingly, the breakdown viscosity (BD) decreased significantly in almost all RHMT-treated samples compared with NS (*p* < 0.05), with the lowest value observed in 100-RHMT-7 (1184 cP), suggesting improved resistance of starch granules to thermal and mechanical disruption; notably, 80-RHMT-1 did not differ significantly from NS (Table 1). These changes may be attributed to stronger hydrogen bonding and tighter molecular packing between amylose and amylopectin chains, which enhanced granular rigidity and reduced swelling and breakdown during pasting [20,21,22,23,24]. Structural evidence supports this interpretation. SEM images showed that starch granules treated at 80 and 100 °C largely retained their morphology, with surface roughening and slight aggregation but without severe collapse or fusion, indicating enhanced resistance to shear-induced disintegration during pasting. Although the FTIR 1047/1022 and 995/1022 ratios slightly decreased compared with NS, samples treated at 80 and 100 °C exhibited relatively higher values than those treated at 120 °C, suggesting better preservation of short-range ordered structures under moderate treatment. XRD analysis further confirmed that the A-type crystalline pattern was maintained in all samples. Therefore, the reduced BD is mainly associated with restricted granule swelling and improved structural integrity during thermal and mechanical processing. Along with the generally reduced BD values, the trough viscosity (TV) tended to be higher than that of NS for most RHMTs (*p* < 0.05), particularly at 80 and 100 °C; however, TV for 80-RHMT-1 and 120-RHMT-7 did not differ significantly from NS (Table 1). Notably, although BD was significantly reduced in 120-RHMT-7, TV was not significantly different from NS, suggesting that excessive treatment may partially disrupt the granular structure and offset the increase in TV [25]. TV dropped to 2466 cP in 120-RHMT-7, supporting this interpretation. A similar trend was observed for final viscosity (FV): FV increased significantly after RHMT at 80 °C compared with NS (*p* < 0.05), showed no significant change relative to NS at 100 °C, and increased significantly at 120 °C for 1–5 cycles; however, FV decreased markedly in 120-RHMT-7 (3744 cP), which was significantly lower than NS (*p* < 0.05). These results further indicate partial structural weakening under excessive treatment severity. The setback viscosity (SB) was only slightly affected at 80 °C, with a significant increase observed only for 80-RHMT-1 compared with NS (*p* < 0.05). In contrast, SB decreased significantly at 100 °C for all cycle numbers (*p* < 0.05), and at 120 °C a significant reduction was observed only for 120-RHMT-7 (*p* < 0.05), reaching the lowest value (1278 cP). In general, lower SB values are associated with a reduced short-term retrogradation tendency, probably because restricted swelling and reduced amylose leaching limit molecular reassociation during cooling [5,23]. However, 120-RHMT-7 not only showed the lowest SB but also exhibited pronounced reductions in PV and FV. SEM observations further revealed evident granular disruption in this sample. These results indicate structural deterioration under excessive treatment severity. Therefore, the lower SB in 120-RHMT-7 may partly result from structural damage rather than improved functional stability. In contrast, samples treated at 100 °C showed a clear reduction in SB while maintaining FV comparable to NS and relatively intact granule morphology, suggesting a more balanced modification effect. Overall, RHMT significantly altered the pasting behavior of hard proso millet starch by increasing PT and often increasing TV, while generally decreasing PV and BD; SB decreased most consistently at 100 °C and under the most severe condition (120-RHMT-7). Treatment at 100 °C with multiple cycles appeared to be the most effective in improving paste stability and reducing retrogradation tendency without the partial structural weakening observed under the most severe 120 °C treatment.

### 3.2. Rheological Properties

#### 3.2.1. Dynamic Rheological Analysis

**Figure 1:**
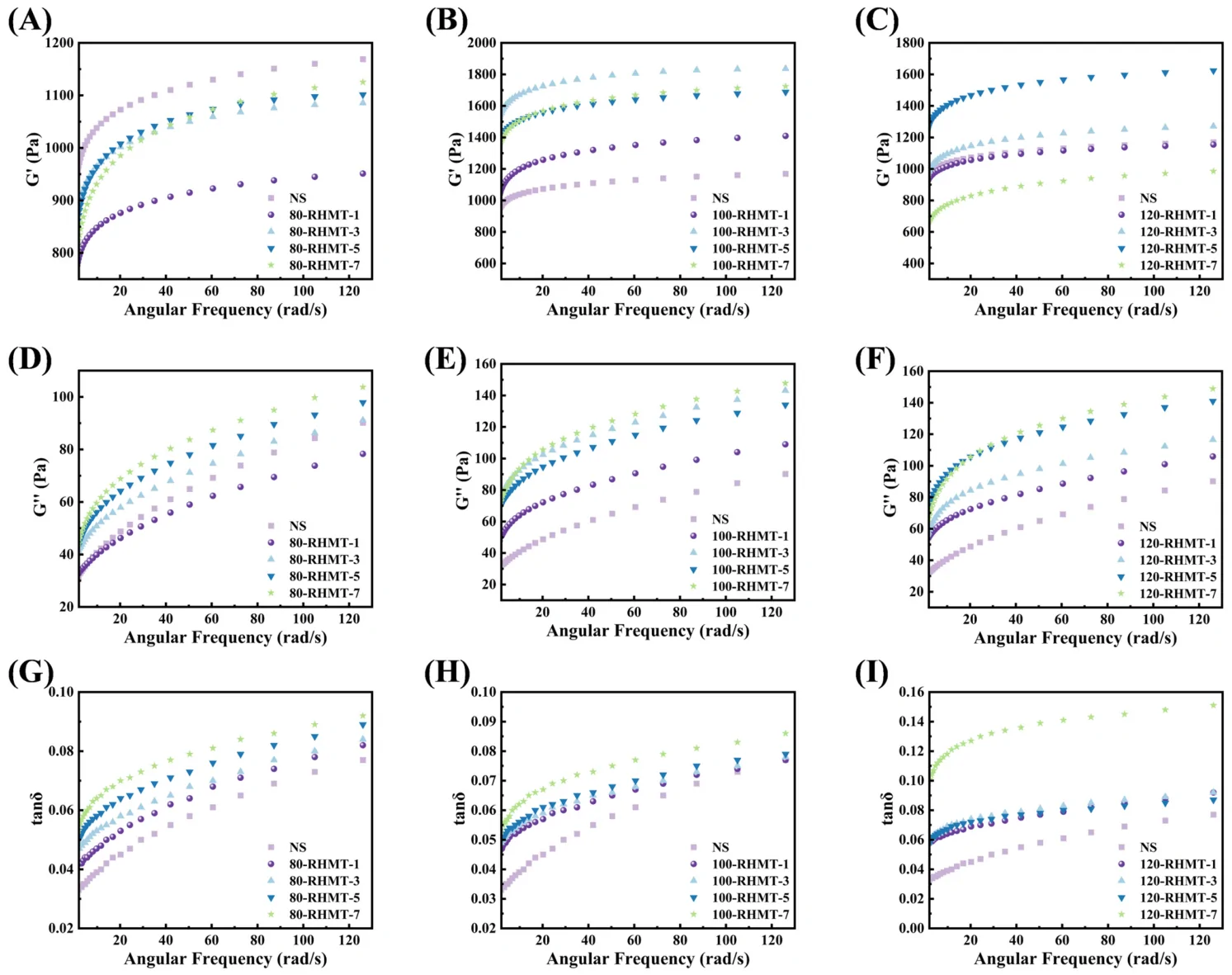
Dynamic rheological properties of native starch (NS) and starch modified by repeated heat–moisture treatment (RHMT) at varying temperatures (80 °C, 100 °C, and 120 °C). (**A**–**C**) storage modulus (*G′*); (**D**–**F**) loss modulus (*G″*); (**G**–**I**) loss factor (tan *δ*).

Dynamic oscillatory frequency sweeps were employed to characterize the viscoelastic behavior of the starch gels, as shown in Figure 1. The rheological profiles revealed a predominantly elastic behavior for all gels, where the storage modulus (*G′*) was substantially greater than the loss modulus (*G″*) over all tested frequencies (tan *δ* < 0.15). In addition, both *G′* and *G″* increased with angular frequency, suggesting the formation of relatively stable gel networks. RHMT markedly affected the viscoelastic behavior of the starch gels, and in general, *G′* increased after treatment but declined under more severe conditions. Among the treated samples, starch gels prepared at 100 °C showed higher *G′* values than those treated at 80 °C and 120 °C, indicating that this condition was more favorable for reinforcing the gel network. This observation aligns with the findings of Ambigaipalan et al. [26], who suggested that heat–moisture treatment induces strong amylose-amylopectin interactions. These interactions are thought to restrict the mobility of starch chains within the amorphous regions, thereby strengthening the macroscopic network and enhancing the elastic properties of the gel [26]. We postulate that this enhanced elasticity is likely associated with the reorganization of short-range molecular order and strengthened intermolecular forces. However, when treatment intensity was further increased, especially in the 120-RHMT-7 sample, *G′* decreased noticeably, implying that excessive RHMT may partially weaken the network structure formed during gelation. A similar trend was also observed for *G″*, indicating that both the elastic and viscous components of the gel were influenced by treatment severity. Tan *δ* (*G″*/*G′*) reflects viscoelastic character, with higher values indicating greater fluidity [27]. As shown in Figure 1, the tan *δ* values of the RHMT-treated samples were generally higher than that of NS, suggesting a relative increase in the viscous contribution over the elastic response following modification. Notably, the 120-RHMT-7 sample exhibited the highest tan *δ* value alongside a relatively low *G′*. This confirms that excessive treatment conditions weakened the internal gel network, resulting in a structure with diminished elasticity. These findings align with previous studies [27,28], suggesting that the degradation of the starch network leads to a softer gel texture. This is consistent with the significantly reduced SB of the 120-RHMT-7 sample, confirming that RHMT lowers gel strength but effectively enhances resistance to retrogradation. Overall, these results indicate that RHMT effectively modified the viscoelastic properties of hard proso millet starch gels. Moderate treatment, particularly at 100 °C, enhanced gel strength and elasticity, whereas excessive treatment at 120 °C with multiple cycles tended to weaken the gel network. This trend was also consistent with the changes observed in RVA parameters, especially FV and TV, further confirming that RHMT-induced structural rearrangement played an important role in determining starch gel behavior.

#### 3.2.2. Static Rheological Analysis

##### Apparent Viscosity

**Figure 2:**
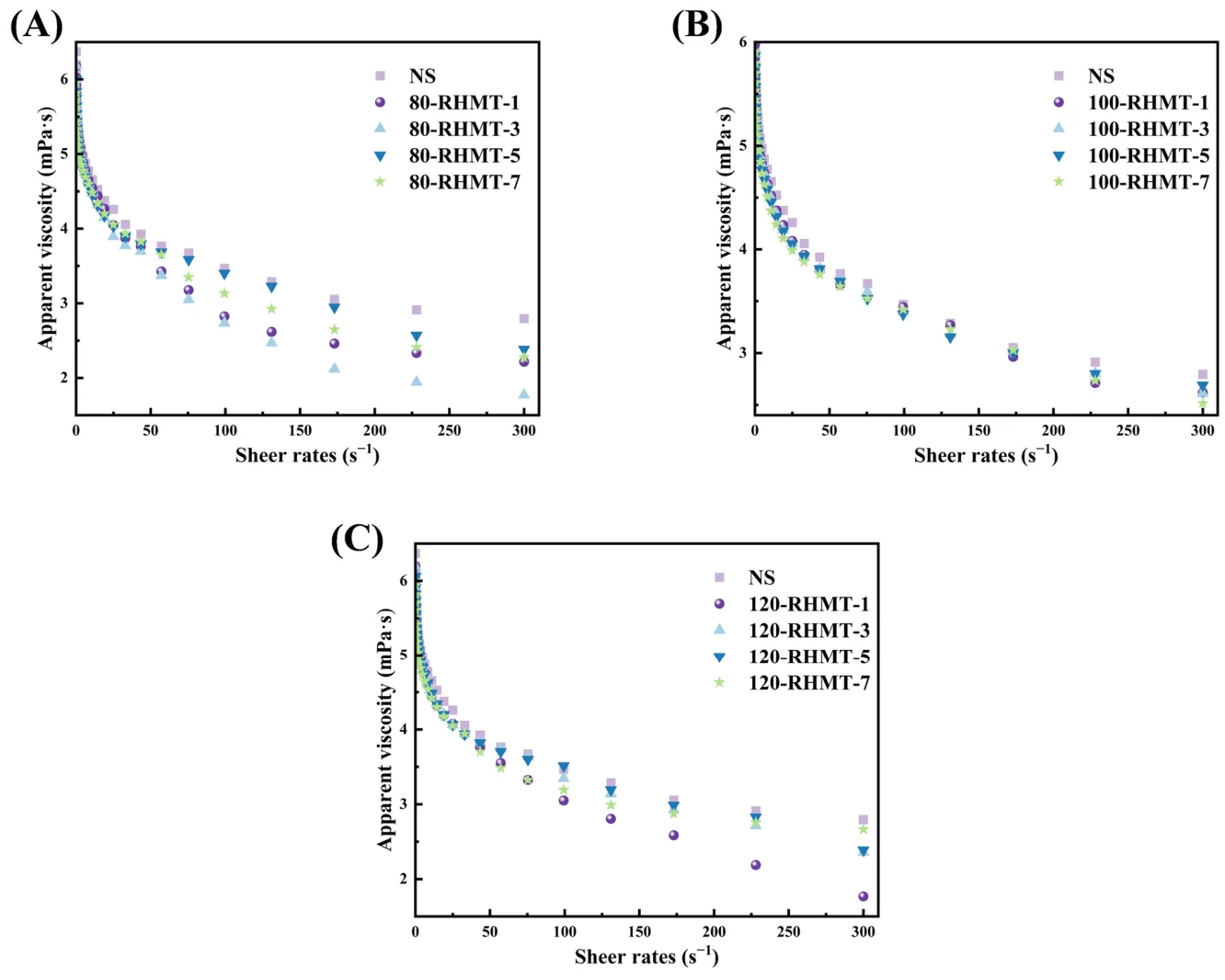
Apparent viscosity of native starch (NS) and starch modified by repeated heat–moisture treatment (RHMT) at varying temperatures. (**A**) 80 °C, (**B**) 100 °C, (**C**) 120 °C.

As shown in Figure 2, the apparent viscosity of all starch pastes decreased rapidly with increasing shear rate, exhibiting typical shear-thinning (pseudoplastic) fluid behavior. In the gelatinized starch system, starch molecular chains entangle with each other. However, as the shear rate increases, the number of molecular entanglements decreases, causing the system to exhibit shear-thinning behavior. Compared with NS, the apparent viscosity of RHMT-modified starches was generally lower across the entire shear rate range. This reduction suggests that the thickening capacity of hard proso millet starch is significantly diminished by hydrothermal treatment. The restricted swelling of starch granules, as evidenced by the lower PV, decreases the effective volume fraction of the dispersed phase within the continuous medium. Consequently, the reduced inter-granular friction and collision frequency lead to a substantial decrement in the overall flow resistance and the internal viscous drag of the slurry [29].

As listed in Table 3, the high coefficients of determination (*R*^2^ > 0.95) across all samples confirm the model’s reliability in characterizing the rheological properties. According to the two-way ANOVA (Table 2), *K* and *n* were significantly affected by the number of cycles (*C*) and the *T* × *C* interaction (*p* < 0.001), indicating that the effects of RHMT cycles depended on the treatment temperature. All samples exhibited a flow behavior index (*n*) of less than 1, which is indicative of typical pseudoplastic behavior [30]. Compared with NS, RHMT increased *n* significantly for all treatments (*p* < 0.05; Table 3), suggesting a reduced degree of shear-thinning behavior, likely due to strengthened granular rigidity. Notably, the main effect of temperature on *n* was not significant (*p* = 0.576; Table 2), implying that changes in *n* were mainly governed by cycle number and the temperature–cycle combination rather than temperature alone.

Conversely, the consistency coefficient (*K*), which reflects the overall thickness of the fluid, decreased significantly after RHMT compared with NS (*p* < 0.05; Table 3) and varied with treatment severity, with the lowest *K* observed at 120-RHMT-7. The observed rheological thinning, in agreement with the viscosity profiles presented in Figure 2, stems mainly from the impaired swelling capacity of starch granules after the RHMT process. This limitation on granular expansion effectively decreases the volume fraction of the dispersed phase, thereby weakening the inter-granular interactions and ultimately lowering the system’s overall viscosity [13,31].

##### Creep and Recovery Analysis

**Figure 3:**
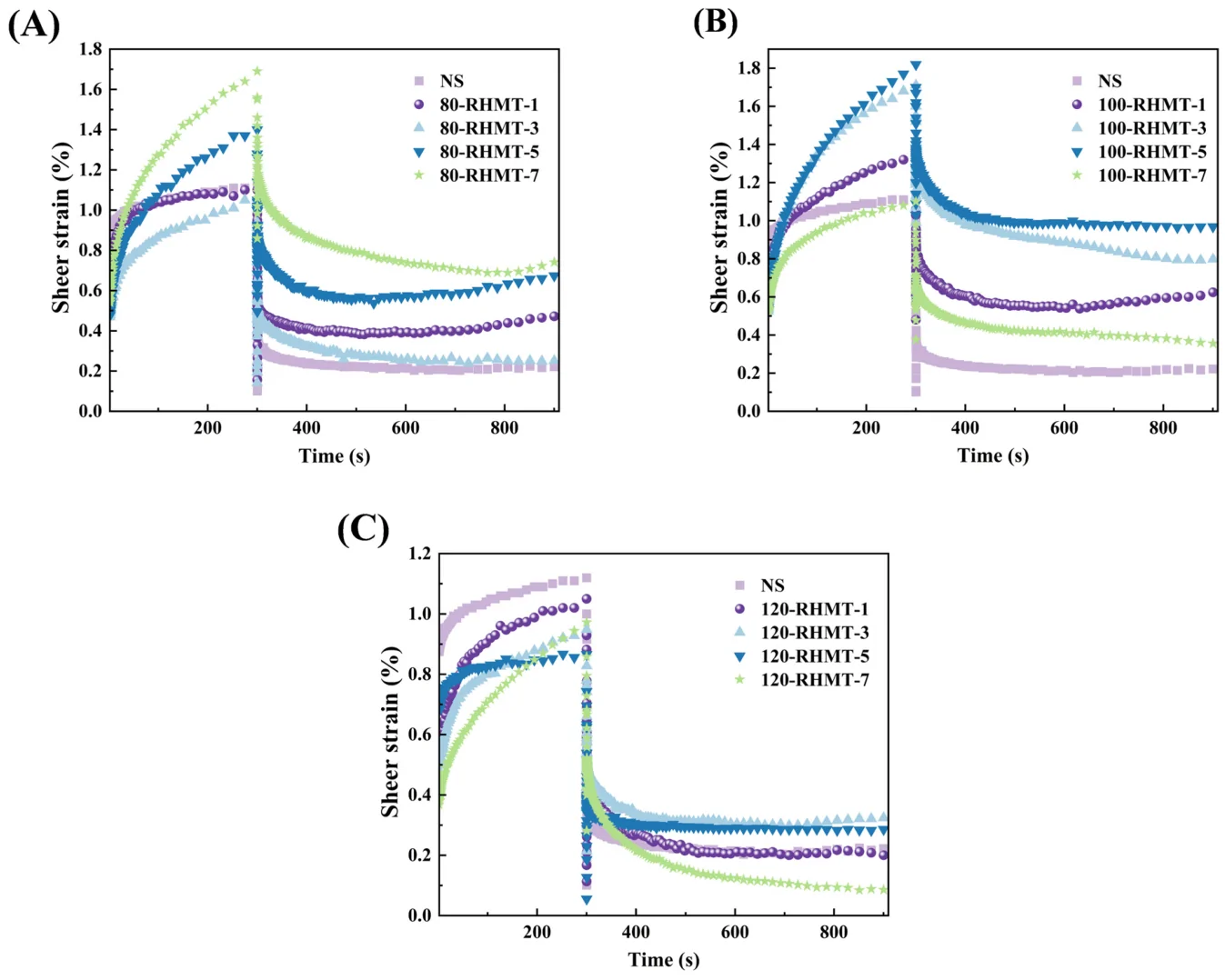
Creep and recovery behavior of native starch (NS) and starch modified by repeated heat–moisture treatment (RHMT) at varying temperatures. (**A**) 80 °C, (**B**) 100 °C, (**C**) 120 °C.

Creep–recovery tests were conducted to evaluate the viscoelastic deformation behavior and internal structural strength of the starch gels under constant stress. The creep phase reflects material rigidity and flow resistance, whereas the recovery phase represents elastic resilience. As shown in Figure 3, the strain of all samples increased rapidly upon stress application (instantaneous elastic response), followed by a gradual increase (retarded elastic and viscous response), indicating molecular rearrangement within the gel network [32,33]. After stress removal, the strain partially recovered but did not return to zero, leaving a permanent deformation. In general, a higher maximum creep strain indicates a softer gel structure with lower rigidity. As illustrated in Figure 3A,B, most RHMT-treated samples, particularly those treated at 80 °C and the initial cycles at 100 °C, exhibited higher creep strains and larger irrecoverable deformations than NS, suggesting reduced gel rigidity. This behavior is consistent with the restricted granule rupture (low BD) observed during pasting, where intact granules contribute less to network reinforcement. However, 100-RHMT-7 showed a significantly lower creep strain than NS (Figure 3B), indicating enhanced resistance to deformation. This result, together with its highest PT and lowest BD values, suggests strengthened granular interactions within the gel matrix, allowing granules to function as rigid fillers that resist applied stress [33]. It should be noted that such macroscopic gel rigidity mainly reflects particle–matrix interactions rather than molecular-scale structural rearrangement alone. Therefore, increased stiffness does not necessarily imply a proportional increase in slowly digestible starch formation. In contrast, 120-RHMT-7 exhibited the lowest overall strain response (Figure 3C). This behavior may be attributed to extensive structural rearrangement and reduced water-holding capacity under excessive treatment, resulting in a compact network with restricted molecular mobility rather than genuinely enhanced structural integrity.

Creep data were further fitted using the Burgers model, and the obtained parameters (Table 4) quantitatively supported these observations. Compared with NS, all RHMT-treated samples showed reduced *J*_1*C*_ values, indicating decreased delayed elastic compliance. The instantaneous compliance (*J*_0*C*_) exhibited a treatment-dependent variation, with decreases at lower treatment intensity (e.g., 80-RHMT-1 and 100-RHMT-1) but increases at prolonged cycles, whereas 120-RHMT-7 showed a pronounced reduction (0.85 vs. 1.53 for NS). In terms of viscous behavior, *μ*_0_ decreased in most samples treated at 80 °C and during the early cycles at 100 °C, but markedly increased at higher temperature and prolonged treatment, particularly for 100-RHMT-7 (7.7974) and 120-RHMT-7 (8.50), consistent with their enhanced resistance to deformation. The relatively high *R*^2^ values (0.90–0.98) confirmed the reliability of the model fitting. Two-way ANOVA (Table 2) further revealed that temperature significantly affected *J*_0*C*_ (*p* = 0.024), *λ*_1*C*_ (*p* = 0.043), and especially *μ*_0_ (*p* < 0.001), whereas cycle number and the interaction effect were not significant (*p* > 0.05), indicating that treatment temperature played a dominant role in modulating gel viscoelasticity.

### 3.3. In Vitro Digestion Properties

**Figure 4:**
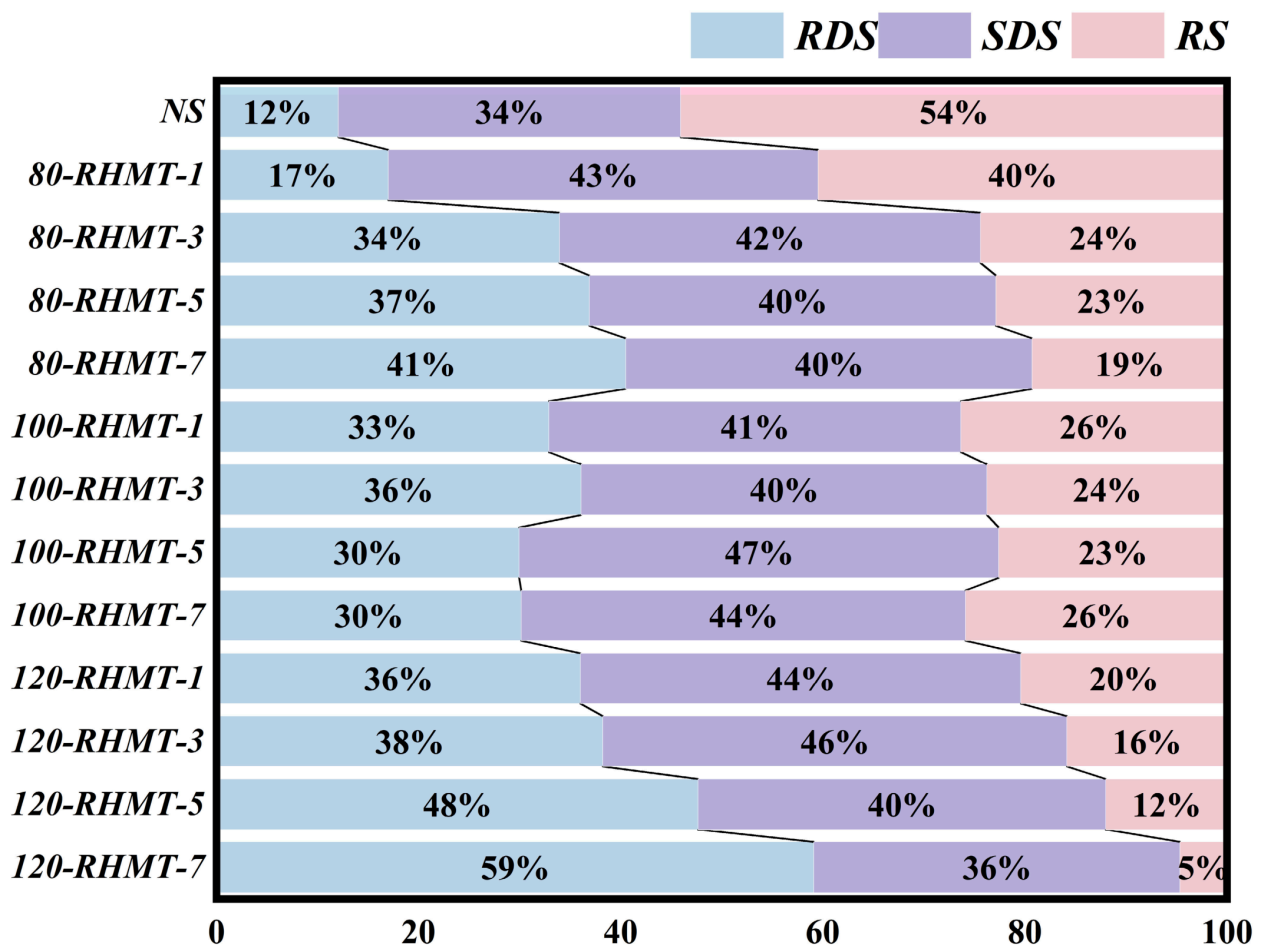
Contents of rapidly digestible starch (RDS), slowly digestible starch (SDS), and resistant starch (RS) in native starch (NS) and starch modified by repeated heat–moisture treatment (RHMT) at varying temperatures. (80 °C, 100 °C, and 120 °C).

The proportions of the starch fractions are presented in Figure 4. Compared with NS (which had a high RS of 54%), the RS content of the modified starches generally decreased after RHMT (varying between 5% and 40%). In contrast, the contents of RDS and SDS increased significantly (*p* < 0.05, Table S1). Notably, SDS increased after RHMT, with 100-RHMT-5 reaching the highest value (≈47%), representing a 38.2% increase compared to NS. Earlier investigations indicated that HMT induces structural reorganization, which may promote the growth of crystalline regions alongside less perfect structural domains [16]. Therefore, the substantial conversion from RS to SDS under moderate RHMT (100 °C) is likely associated with such structural rearrangement, creating a denser and more rigidified internal granular architecture (which is indirectly reflected by the macroscopic gel strengthening upon gelatinization, i.e., the higher *G′* and TV in earlier rheological data). This reinforced granular matrix presumably acts as a steric hindrance that effectively slows hydrolysis without completely blocking enzyme access. Although total RS decreased, this shift is functionally valuable: native Type 2 RS is generally heat-labile during cooking [4], whereas the RHMT-induced SDS fraction provides a gradual in vitro digestion profile, a structural-functional relationship that is further validated by our multivariate PCA results (Section 3.6.2). On the other hand, as treatment severity further increased (specifically at 120-RHMT-7), the SDS content declined compared with 100-RHMT-5, while the RDS content surged to its maximum (59%) and RS dropped to a minimum (5%). This drastic change is attributed to severe granular degradation and loss of structural integrity caused by excessive heating. As the internal granular structure collapsed (consistent with the sharp drop in *G′* and TV), the structural integrity that previously hindered digestive enzymes was largely compromised, making the starch highly susceptible to rapid digestion. This extreme structural disruption is robustly supported by the thermodynamic collapse (Δ*H*) observed in the subsequent DSC analysis (Section 3.5.1). Therefore, within the tested range, RHMT at 100 °C appears to be an optimal condition for increasing SDS content.

### 3.4. LOS Plot Analysis and CPS Kinetic Model Fitting

**Figure 5:**
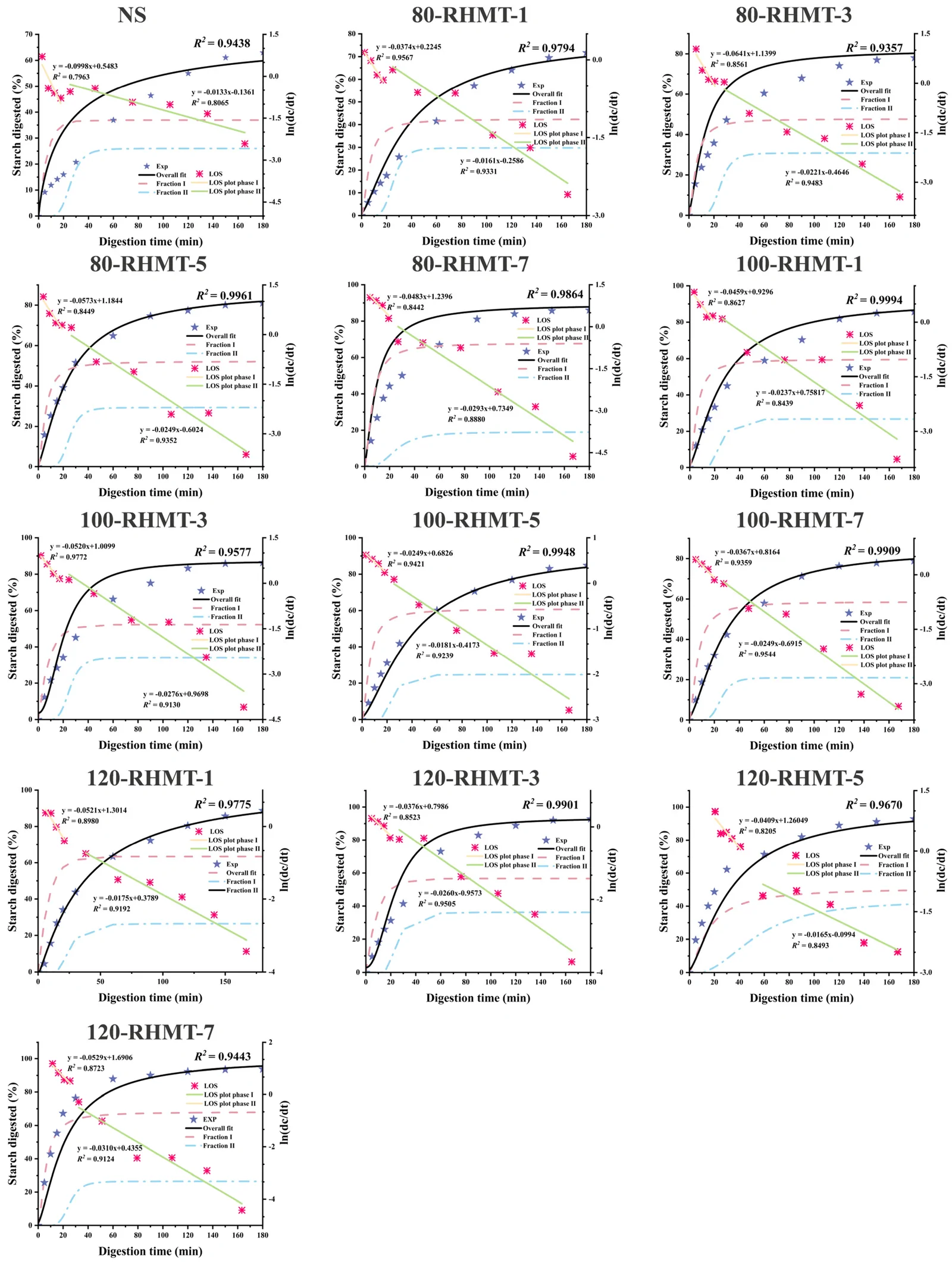
LOS and CPS kinetic model fitting of native starch (NS) and starch modified by repeated heat–moisture treatment (RHMT) at varying temperatures. (80 °C, 100 °C, and 120 °C). Fraction I, the rapidly digested part. Fraction II, the slowly digested part. Exp, experimental data.

To analyze the enzymatic digestion characteristics of different fractions, the LOS plot (Figure 5) was first used. It can distinguish digestible fractions with different structures in the system. The results showed the LOS plots of all starch samples under different RHMTs. All plots exhibited two discontinuous linear segments. This indicates a two-phase digestion behavior for the starches. The digestion process includes a rapidly digestible fraction (Fraction I) and a slowly digestible fraction (Fraction II) [34].

Based on this two-phase behavior, this study used the CPS digestion kinetic model to fit the data. The CPS fitting curves (also shown in Figure 5) showed a clear trend. The fast and slow digestion processes were not completely independent. They shared a certain overlapping period [35]. As shown in Figure 5, the rapid fraction approached its maximum extent of digestion (*C*_1∞_) within the first 20 min. During this time, the slow digestion fraction also initiated. The slow fraction then dominated the digestion process. The whole system reached a final plateau at about 120 min. This CPS kinetic pattern can be explained by the protective layer mechanism proposed by Li and Hu [35]. The rapid digestion fraction acts like an outer shell. It wraps around the inner slow digestion matrix. The enzymes must destroy this outer shell first. After that, the inner slow digestion fraction can fully contact the enzymes. For the 100-RHMT treated samples, it is speculated that the structural ordering delayed the destruction of this outer shell, which in turn further protected the SDS fraction.

Table 3 lists the digestion rate constants (*k*_1_ and *k*_2_) and the maximum digestion extents (*C*_1*∞*_ and *C*_2_*_∞_*). These parameters were obtained from CPS fitting, with high goodness of fit across samples (*R*^2^ mostly > 0.97). According to the two-way ANOVA (Table 2), *k*_1_, *C*_1*∞*_, *C*_2_*_∞_*, and *RC* were significantly affected by temperature (*T*), number of cycles (*C*), and their interaction (*T* × *C*), whereas *k*_2_ was mainly influenced by *C* and *T* × *C*. In all samples, the rapid digestion rate (*k*_1_) was generally higher than the slow digestion rate (*k*_2_), which matches normal starch digestion patterns [36]. NS had a *k*_1_ value of 0.0998 min^−1^. Compared to NS, *k*_1_ decreased significantly in all treated groups (*p* < 0.05; Table 3). Notably, the *k*_1_ and *k*_2_ values were very close in 100-RHMT-5 (*k*_1_ = 0.0202 min^−1^; *k*_2_ = 0.0201 min^−1^), indicating that the initial rapid digestion stage was strongly inhibited. This is likely because the 100 °C hydrothermal environment promoted potential structural reorganization to form partially ordered structures and a strengthened macroscopic gel network, presumably creating steric hindrance that limited initial enzyme access. Under this condition, the reduction in *k*_1_ and the narrowed gap between *k*_1_ and *k*_2_ suggest a relatively stronger delay of early-stage digestion and a tendency toward slower digestion behavior. In contrast to the consistent reduction in *k*_1_, the maximum digestion extents (*C*_1*∞*_ and *C*_2_*_∞_*) showed a more complex, *T* × *C*-dependent response (Table 2 and Table 3). The rapid digestion extent (*C*_1*∞*_) of NS was 38.38%. After RHMT, *C*_1*∞*_ increased under several conditions (e.g., 80-RHMT-7 and 120-RHMT-7, both ≈ 67%), indicating that severe treatment could disrupt granule structure and expose more readily digestible components. Conversely, 120-RHMT-3 showed a significantly lower *C*_1*∞*_ (31.26%) but a markedly higher *C*_2_*_∞_* (61.59%), implying a redistribution from the rapid to the slow digestion phase rather than a uniform increase in the rapid digestible fraction. These results are consistent with the pasting and rheological analyses, suggesting that RHMT modified the balance between rapid and slow digestion fractions depending on the combined temperature–cycle condition.

### 3.5. Structural Properties

#### 3.5.1. Thermal Properties

The thermal transition parameters (*To*, *Tp*, *Te*, Δ*H*) are summarized in Table 5. Compared with native starch (NS), all RHMT-treated samples exhibited progressively higher transition temperatures (*p* < 0.05). This endothermic shift indicates enhanced thermal stability of the starch crystallites, driven by the rearrangement of molecular chains into more thermodynamically stable configurations during repeated thermal cycling. These findings perfectly corroborate the increased PT observed in the RVA profiles. Conversely, the gelatinization enthalpy (Δ*H*), which reflects the energy required to unwind double helices, decreased after RHMT. Under moderate conditions (80 and 100 °C), Δ*H* showed a moderate reduction. This suggests a structural balance where imperfect crystallites partially melted, while the remaining structure reorganized into a stable, rigidified matrix that delayed enzymatic hydrolysis, aligning with the peak SDS formation at 100 °C. However, excessive treatment at 120 °C (e.g., 120-RHMT-7) caused a drastic drop in Δ*H* to 3.25 J/g (vs. 11.51 J/g in NS). This thermodynamic collapse confirms the severe unwinding of double-helical structures and extensive crystalline disruption, fully supporting the granular damage observed in SEM, the loss of crystalline order in XRD, and the corresponding surge in RDS.

#### 3.5.2. XRD

**Figure 6:**
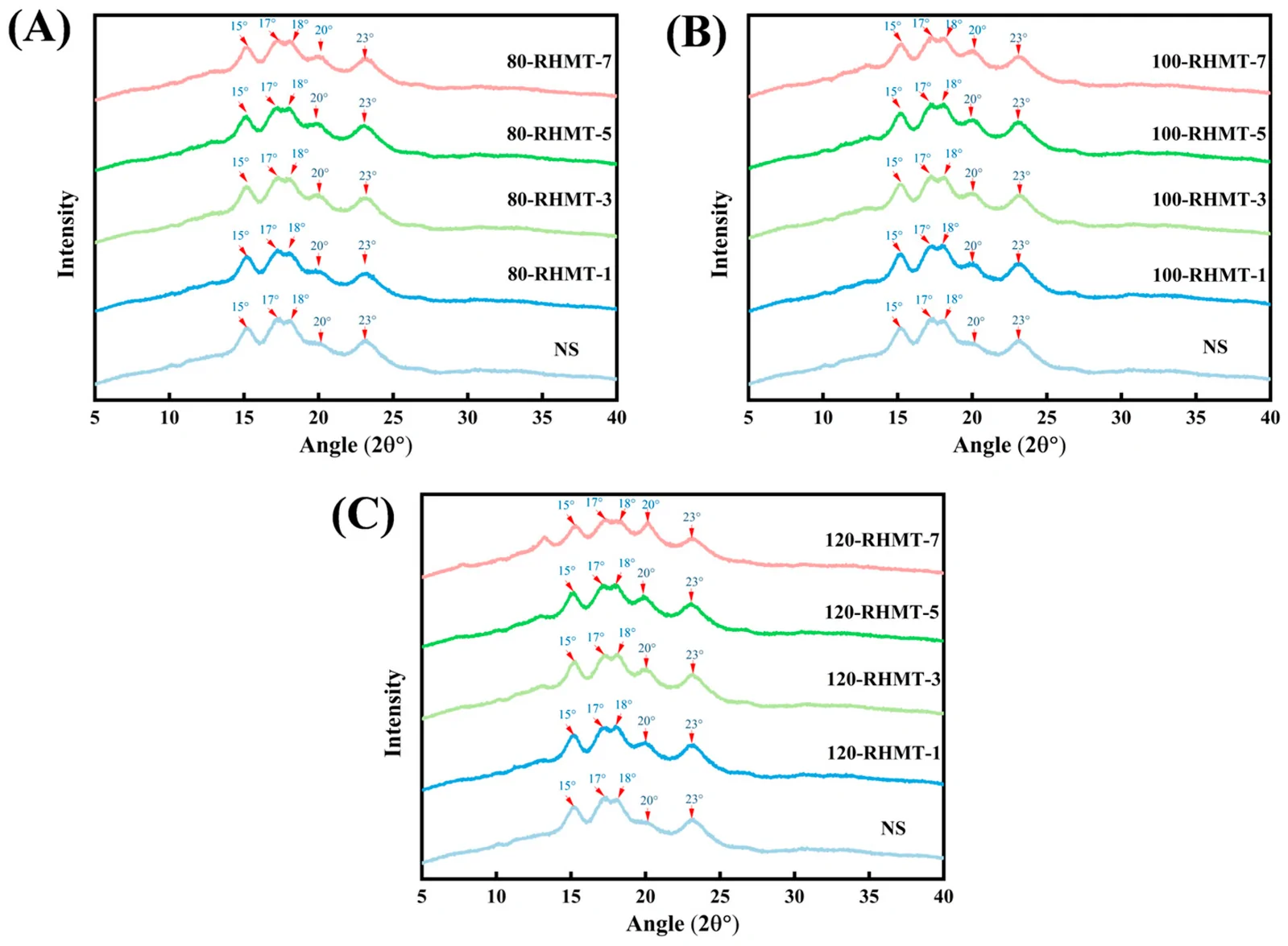
X-ray diffraction patterns of native starch (NS) and starch modified by repeated heat–moisture treatment (RHMT) at varying temperatures. (**A**) 80 °C, (**B**) 100 °C, (**C**) 120 °C.

Figure 6 shows the XRD patterns of all samples. Prominent diffraction peaks were observed for NS at 2*θ* angles around 15°, 17°, 18°, and 23°. This indicates a typical A-type crystal structure. The low-intensity diffraction peak appearing at a 2*θ* value of about 20° can be attributed to the formation of starch-lipid complexes [37]. RHMT did not change the positions of these main peaks. Nevertheless, the XRD profiles of the samples revealed notable changes in the intensity of the main diffraction peaks. This phenomenon indicates that the hydrothermal modification occurred mainly in the amorphous region of the starch [38,39]. The relative crystallinity (*RC*) of starch reflects the degree of order within its internal crystalline structure and serves as a key indicator influencing its digestibility, thermal behavior, and processing performance [24]. As shown in Table 5, the *RC* values of RHMT-treated samples exhibited a fluctuating trend depending on treatment temperature and cycle number, rather than a uniform increase or decrease relative to NS (14.39%). This observation is supported by the two-way ANOVA results (Table 2), where temperature (*T*), number of cycles (*C*), and their interaction (*T* × *C*) all showed significant effects on *RC* (*p* < 0.001), indicating a strong dependence on the combined *T* × *C* condition. A reduction in *RC* was observed under some treatment conditions. For instance, the *RC* of 80-RHMT-7 decreased to 13.04%, corresponding to a 9.4% decline compared with NS (*p* < 0.05). Similarly, 120-RHMT-3 showed a significantly lower *RC* (13.78%) than NS (*p* < 0.05). The repetitive thermal cycling involved in RHMT may partially disrupt double-helical structures and weaken crystalline domains, leading to reduced *RC* values. Similarly, Zhang et al. [40] reported that while RHMT did not alter the crystalline form of wheat starch, it could induce a decrease in *RC* due to structural rearrangement and partial crystalline destabilization. Notably, several samples exhibited *RC* values significantly higher than that of NS (*p* < 0.05), including 80-RHMT-3 (15.83%), 100-RHMT-1 (17.72%), and 120-RHMT-5 (15.60%). This may be attributed to the rearrangement and realignment of starch chains under certain RHMT conditions, where thermal energy enhances molecular mobility and promotes the formation of more ordered crystalline regions [4]. Therefore, RHMT induces a dynamic structural evolution characterized by crystalline reorganization under some *T* × *C* combinations and partial crystalline disruption under others.

#### 3.5.3. FTIR

**Figure 7:**
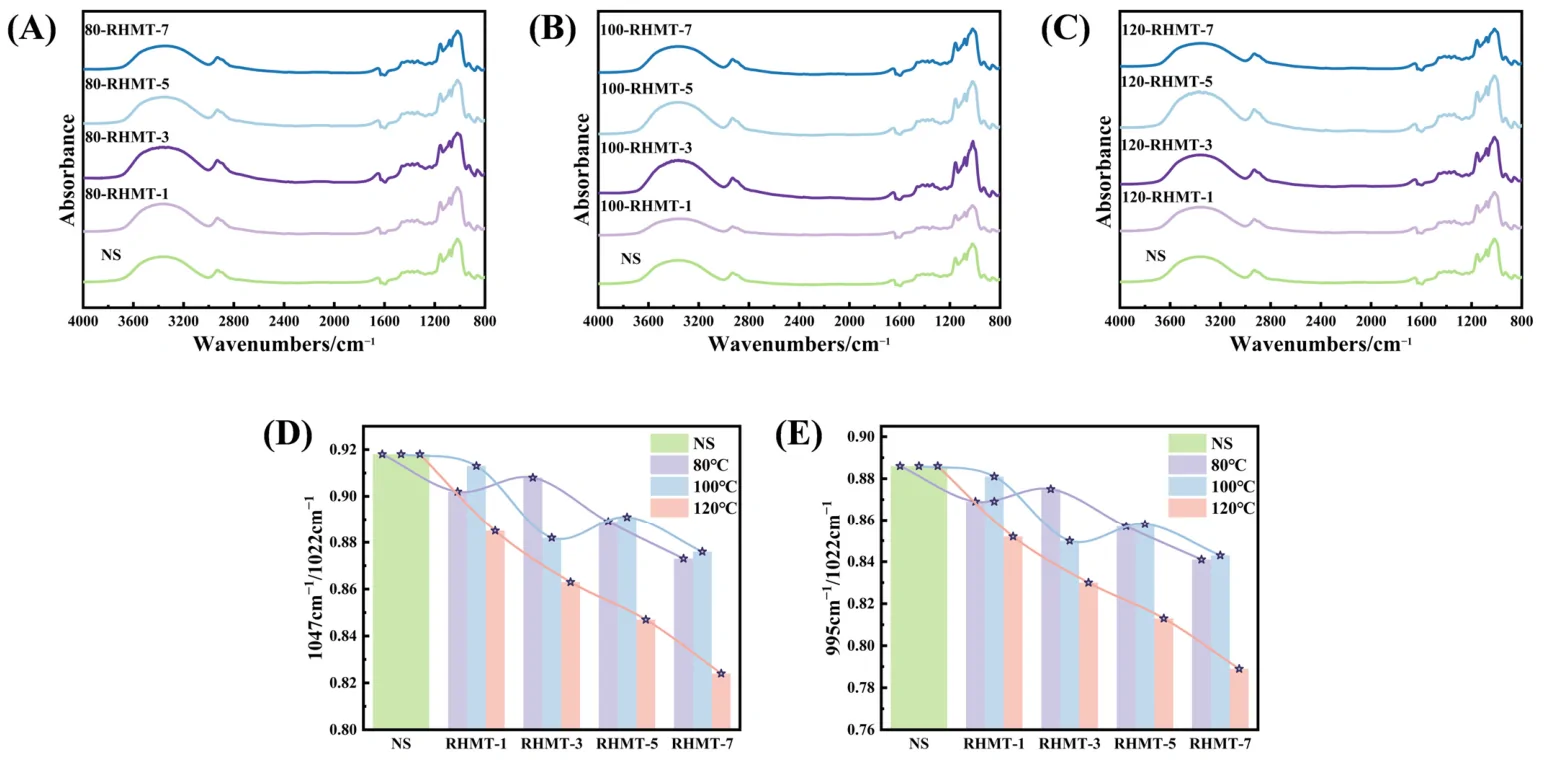
FTIR deconvoluted spectra (4000–800 cm^−1^) of native starch (NS) and starch modified by repeated heat–moisture treatment (RHMT) at varying temperatures. (**A**) 80 °C, (**B**) 100 °C, (**C**) 120 °C. (**D**,**E**) Variation trends of short-range structural parameters. The star symbol is used solely as a data point marker and does not indicate statistical significance.

Figure 7A–C shows the FTIR spectra of the starches. The lack of any additional peaks in the post-RHMT spectra demonstrates that the formation of new covalent linkages or functional groups did not occur. Consequently, the observed changes were attributed to a reorganization of the starch molecular structure. This result agrees with previous reports [6]. To further evaluate changes in short-range structure, the ratios of 1047 cm^−1^/1022 cm^−1^ and 995 cm^−1^/1022 cm^−1^ were analyzed (Figure 7D,E). The 1047 cm^−1^/1022 cm^−1^ ratio is generally associated with the degree of short-range molecular order, whereas the 995 cm^−1^/1022 cm^−1^ ratio is used to indicate the evolution of double-helical packing within the granules. Two-way ANOVA showed that treatment temperature and cycle number (and their interaction) significantly affected both ratios (Table S2, *p* < 0.001). As shown in Figure 7D,E, the 1047 cm^−1^/1022 cm^−1^ and 995 cm^−1^/1022 cm^−1^ ratios of samples treated at 80 and 100 °C were generally higher than those treated at 120 °C. This indicates that moderate thermal treatment favors the preservation of short-range ordered structures in starch. This is likely because appropriate temperatures promote molecular chain rearrangement and hydrogen bond formation, leading to relatively stable local structures [41,42], which is highly consistent with the increased melting temperatures (*Tp*) and moderately retained enthalpy (Δ*H*) observed in the DSC analysis (Section 3.5.1). At higher treatment temperatures (120 °C), the reduction in these FTIR ratios indicates a severe disruption of local structural order and double-helical unwinding. This is further corroborated by the drastic drop in Δ*H* discussed earlier. This loss of structural integrity may facilitate enzyme accessibility to starch chains, a finding that corroborates the data in Section 3.3. Additionally, within each temperature, the ratios did not change monotonically with cycle number despite some significant differences (Table S1), indicating that the RHMT cycle number can be tailored to the target application.

#### 3.5.4. SEM

**Figure 8:**
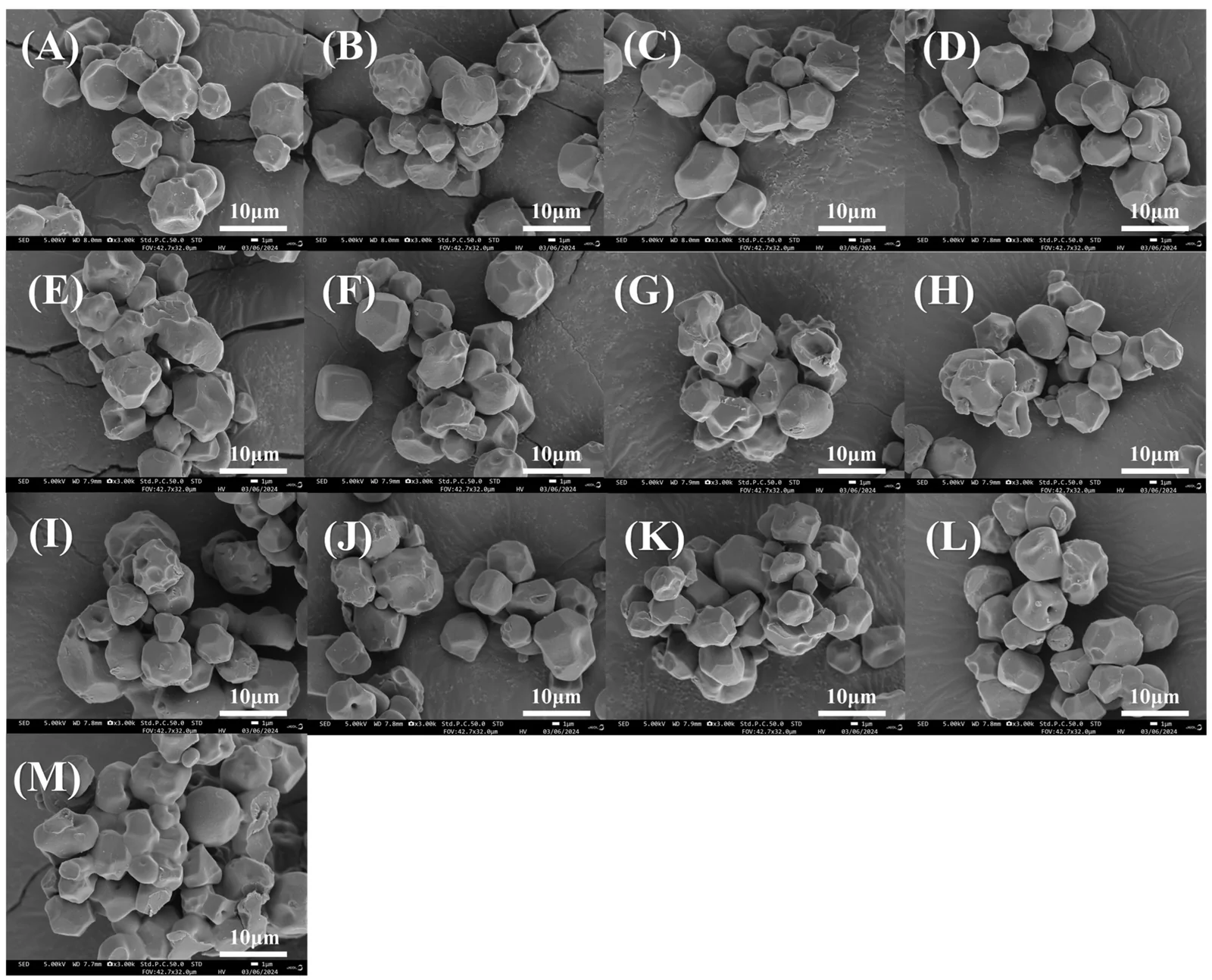
Microscopic images (3000× magnification) of native starch (NS) and starch modified by repeated heat–moisture treatment (RHMT) at varying temperatures. (80 °C, 100 °C, and 120 °C). Samples are labeled as follows: (**A**) NS; (**B**–**E**) 80-RHMT-1, 80-RHMT-3, 80-RHMT-5, 80-RHMT-7; (**F**–**I**) 100-RHMT-1, 100-RHMT-3, 100-RHMT-5, 100-RHMT-7; (**J**–**M**) 120-RHMT-1, 120-RHMT-3, 120-RHMT-5, 120-RHMT-7.

Figure 8 illustrates the microstructural evolution of the starch samples after RHMT. NS granules exhibited polygonal or irregular spherical shapes with relatively smooth surfaces and occasional surface depressions, consistent with previous reports [38]. RHMT induced varying degrees of morphological alteration. To quantitatively complement these visual observations, the average aggregate sizes of the starch samples were evaluated (Table 5). All RHMT-treated samples showed significantly larger aggregate sizes than NS (17.71 ± 0.32 μm, *p* < 0.05), with an overall increasing trend as treatment temperature and cycle number increased (Table 5). At a given temperature, increasing treatment cycles markedly promoted granule aggregation and surface adhesion, leading to the formation of clustered structures [37]. This behavior can be attributed to enhanced molecular mobility and facilitated water diffusion into the amorphous regions under thermal conditions, which promotes partial gelatinization, intermolecular interactions, and structural rearrangement [15]. Consequently, the aggregated granules formed a more compact and dense architecture. For the 100 °C RHMT-treated samples, the disruption was relatively limited, as granules largely retained their overall morphology but exhibited roughened surfaces and pronounced indentations. Such surface modifications, together with enhanced aggregation (aggregate size up to 32.59 ± 0.62 μm for 100-RHMT-7), may reduce enzyme accessibility by acting as a potential steric hindrance [43], which is consistent with the reduced digestibility observed in Section 3.3. In contrast, treatment at 120 °C resulted in severe structural damage. For instance, in the 120-RHMT-7 sample, excessive thermal input led to extensive disruption of the semi-crystalline organization, accompanied by the loss of distinct granular boundaries and the formation of fused aggregates [44]. This structural collapse is consistent with the loss of crystalline order and the increased rapid digestibility discussed earlier, as reflected in its largest aggregate size (36.20 ± 0.67 μm) among all samples.

### 3.6. Statistical Analysis

#### 3.6.1. Hierarchical Clustering and Pearson Correlation Analysis

**Figure 9:**
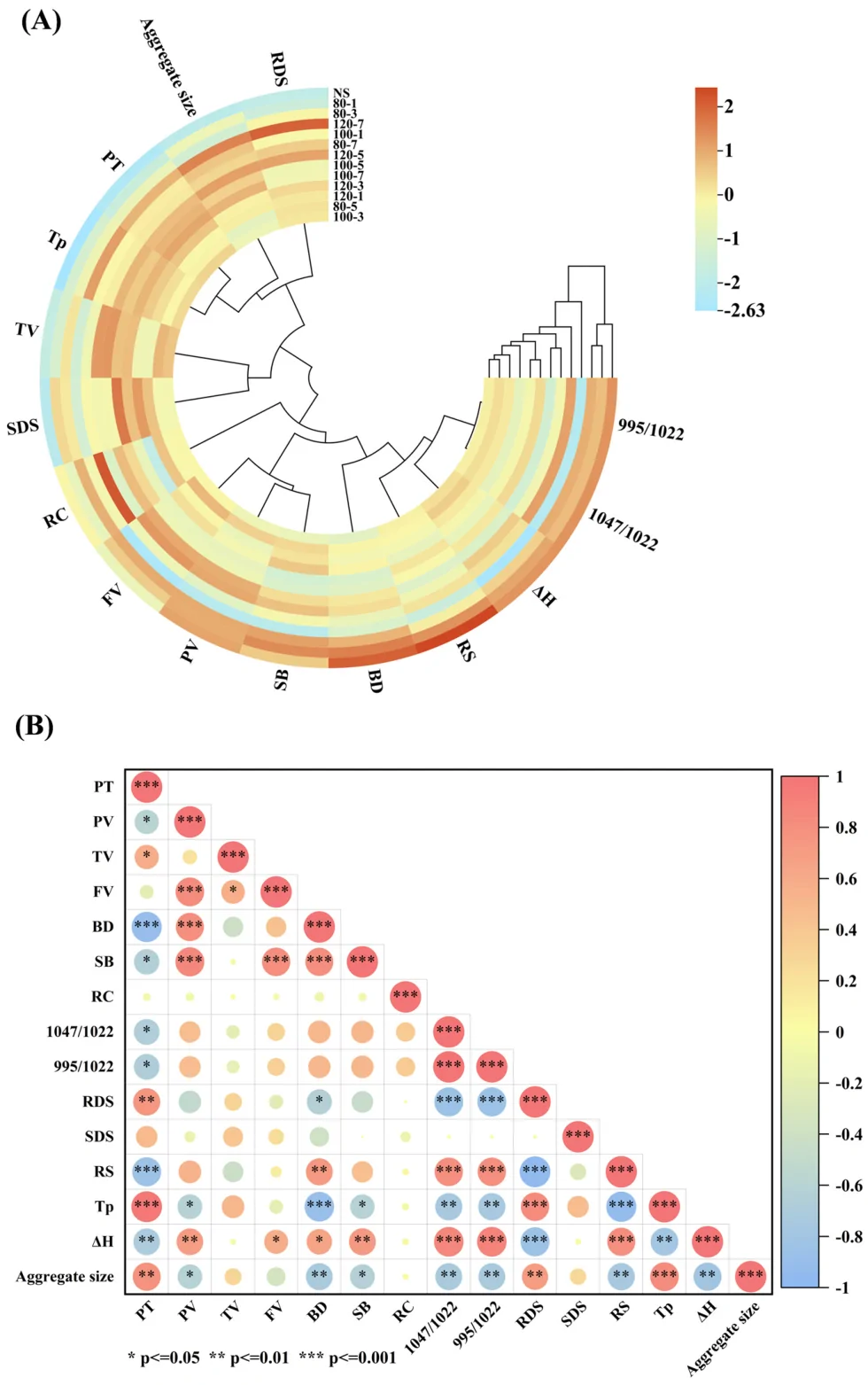

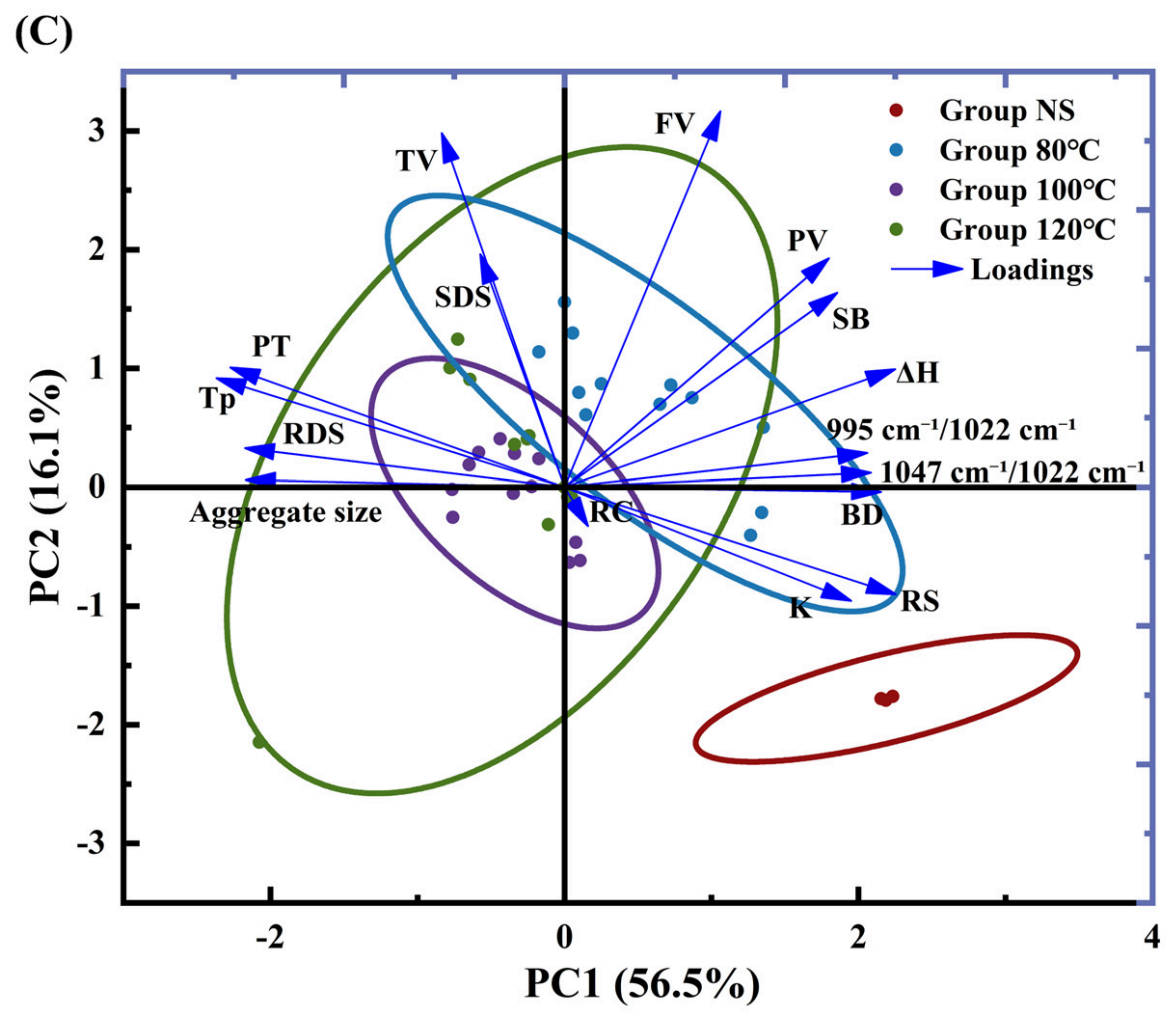
(**A**) Hierarchical clustering heatmap of all starch samples. (**B**) Pearson correlation matrix for the multiscale structural and physicochemical parameters. (**C**) Principal component analysis (PCA) biplot mapping the relationship between sample distribution and key driving variables.

Figure 9A presents the hierarchical cluster analysis heatmap of starches subjected to different RHMT regimes. The samples were primarily grouped according to treatment temperature, indicating that temperature was likely the dominant factor governing variations in physicochemical properties, structural organization, and digestibility [16]. NS formed an isolated cluster characterized by the highest RS, gelatinization enthalpy (Δ*H*), and short-range order (FTIR ratios). Among the treated samples, the 80 and 100 °C groups formed relatively distinct clusters with pronounced SDS-related features. In contrast, the 120-treated samples (particularly 120-RHMT-7) were distinguished by more severe structural deterioration, exhibiting the highest aggregate size, *Tp*, and RDS content.

The Pearson correlation matrix (Figure 9B) further revealed the relationships among pasting, structural, thermal, and digestion-related parameters. Strong internal correlations were observed among the thermal and pasting parameters. Specifically, PT and *Tp* were significantly positively correlated with aggregate size (*p* < 0.01) but negatively correlated with PV, BD, and SB (*p* < 0.05). This suggests that starch samples with severe granular aggregation and higher thermal resistance generally exhibited restricted swelling and reduced viscosity breakdown during heating [45]. In contrast, PV, BD, and SB exhibited strong positive correlations with each other (*p* < 0.001), while FV was significantly positively correlated with PV and SB (*p* < 0.001) [45]. These relationships reflect their potential interdependence in relation to granule swelling and paste stability during the heating and cooling phases.

Regarding molecular structure, a strong positive correlation (*p* < 0.001) was observed between the FTIR structural ratios (1047/1022 and 995/1022 cm^−1^) and Δ*H*. This association suggests that the evolution of short-range molecular order is closely linked to the preservation or unwinding of double helices [16]. Notably, relative crystallinity (*RC*) showed virtually no significant correlation with digestion or other structural parameters. This lack of correlation implies that the functional and digestive properties of RHMT-modified starch might be driven more by double-helical content (Δ*H*) and macroscopic network properties rather than long-range crystalline order alone.

In terms of starch digestibility, RS was significantly positively correlated with FTIR ratios, Δ*H*, and BD (*p* < 0.001), suggesting that preserving native double-helical structures and short-range order is potentially essential for digestion resistance [16,46]. Conversely, RDS was strongly positively correlated with aggregate size, PT, and *Tp* (*p* < 0.01), indicating that thermal disruption and granular aggregation may render the starch more susceptible to rapid enzymatic hydrolysis. In addition, RS was significantly negatively correlated with RDS (*p* < 0.001) [16,46], reflecting the reciprocal relationship between the rapidly digestible and resistant starch fractions. Importantly, SDS showed no significant correlation with native structural parameters (FTIR or Δ*H*) but exhibited positive associations with TV and FV. This observation supports the hypothesis that SDS formation is likely not determined solely by initial crystallinity, but may be governed by a more complex structural balance, such as a robust, rigidified gel network (promoted at 100 °C) acting as a physical barrier to continuously delay enzyme access.

#### 3.6.2. Multivariate Analysis (PCA)

To further elucidate the complex multivariable relationships among the structural, rheological, and digestive properties of the starches, Principal Component Analysis (PCA) was conducted [10]. As shown in Figure 9C, PC1 and PC2 cumulatively accounted for 72.6% of the total variance. The biplot reveals distinct temperature-dependent clustering. NS is completely isolated from the treated groups. Notably, the 100 °C group forms a highly compact cluster, indicating a stable and consistent structural modification. In contrast, the 120 °C samples display a broad dispersion along the negative PC1 axis, reflecting heterogeneous structural deterioration driven by excessive thermal input. Crucially, the loading vectors (blue arrows) directly identify the structural drivers for each digestion fraction. RS aligns positively with PC1, strongly grouping with short-range order (FTIR ratios), double-helical enthalpy (Δ*H*), and breakdown viscosity (BD). This confirms that preserving native crystalline and short-range order is essential for digestion resistance. Conversely, RDS is loaded on the negative PC1 axis, correlating acutely with aggregate size, PT, and *Tp*. This multivariable alignment validates that severe granular aggregation and thermal disruption (prevalent at 120 °C) render the starch highly susceptible to rapid enzymatic hydrolysis. SDS diverges significantly from both RS and RDS, projecting primarily along PC2 towards the 100 °C cluster. SDS exhibits a strong directional correlation with trough (TV) and final viscosities (FV). This suggests that SDS formation is likely governed by the strengthening of the internal granular architecture (promoted at 100 °C). This robust intra-granular structure, which concurrently manifests as high paste stability (TV and FV) upon gelatinization, may act as a physical barrier to continuously delay enzyme access.

## 4. Conclusions

This study systematically investigated the multi-scale structural and functional evolution of hard proso millet starch during RHMT at 80, 100, and 120 °C. Results demonstrated that precise temperature control is critical for tailoring starch properties. Treatment at 100 °C induced favorable structural reorganization and better preserved short-range order, double-helical organization (supported by moderately retained Δ*H*), and granule integrity compared with 120 °C. These structural adaptations directly improved pasting stability and reinforced the macroscopic viscoelastic gel network. As suggested by multivariate analysis, the rigidified internal granular architecture (which positively correlated with the strengthened gel network) likely contributed to delayed enzymatic hydrolysis, reducing the initial digestion rate (*k*_1_) and maximizing the slowly digestible starch (SDS) content to 47.44% in the 100-RHMT-5 sample. In contrast, excessive treatment at 120 °C led to severe loss of structural order and granular collapse. This deterioration was characterized by extensive double-helical unwinding, robustly evidenced by a drastic drop in gelatinization enthalpy (Δ*H*). This profound loss of structural integrity compromised paste stability and rheological elasticity, while also rendering the starch highly susceptible to enzymatic hydrolysis. Consequently, the rapidly digestible starch (RDS) surged to 59%, accompanied by a marked decrease in resistant starch (RS). Overall, the 100 °C RHMT effectively yields SDS-enriched hard proso millet starch, serving as a promising ingredient for the development of starch-based foods with slower in vitro digestion. However, further studies involving specific food matrices, thermal processing (gelatinization), and in vivo glycemic responses are necessary to verify whether this SDS profile is retained in actual cooked products and to confirm its practical functionality.

## Figures and Tables

**Table 1:** Pasting properties of native starch and starch modified by repeated heat–moisture treatment at varying temperatures.

Samples	PT (°C)	PV (cP)	TV (cP)	FV (cP)	BD (cP)	SB (cP)
NS	78.05 ± 0.44	5201 ± 44.09	2348 ± 58.07	4762 ± 6.81	2854 ± 70.00	2410 ± 49.17
80-RHMT-1	80.22 ± 0.51 ^aA^*	5138 ± 205.08 ^aA^	2533 ± 164.15 ^aA^	5339 ± 165.45 ^aA^*	2606 ± 77.66 ^aA^	2807 ± 51.01 ^aA^*
80-RHMT-3	82.33 ± 0.06 ^bA^*	5155 ± 86.56 ^aA^	2940 ± 19.66 ^bAB^*	5598 ± 72.53 ^bcA^*	2216 ± 94.56 ^bA^*	2658 ± 76.62 ^abA^
80-RHMT-5	83.53 ± 0.45 ^cA^*	4874 ± 54.80 ^aA^*	3148 ± 108.82 ^bcA^*	5476 ± 80.58 ^abA^*	1726 ± 127.81 ^cA^*	2328 ± 128.24 ^cA^
80-RHMT-7	83.82 ± 0.49 ^cA^*	5172 ± 223.51 ^aA^	3275 ± 93.66 ^cA^*	5741 ± 117.96 ^cA^*	1897 ± 189.36 ^bcA^*	2466 ± 155.58 ^bcA^
100-RHMT-1	83.72 ± 0.49 ^aB^*	4211 ± 111.80 ^aB^*	2757 ± 109.29 ^aB^*	4821 ± 38.55 ^abB^	1455 ± 120.67 ^aB^*	2064 ± 137.27 ^aB^*
100-RHMT-3	84.58 ± 0.46 ^aB^*	4410 ± 166.58 ^aB^*	3086 ± 74.08 ^bA^*	5010 ± 121.96 ^bB^	1324 ± 184.45 ^aC^*	1924 ± 192.12 ^abB^*
100-RHMT-5	85.62 ± 0.03 ^bB^*	4395 ± 105.31 ^aB^*	3098 ± 76.46 ^bA^*	4803 ± 39.50 ^abB^	1297 ± 181.76 ^aB^*	1704 ± 115.00 ^bcB^*
100-RHMT-7	86.13 ± 0.55 ^bB^*	4309 ± 183.30 ^aB^*	3125 ± 93.55 ^bA^*	4736 ± 102.06 ^aB^	1184 ± 173.30 ^aB^*	1611 ± 123.14 ^cB^*
120-RHMT-1	83.23 ± 0.03 ^aB^*	4355 ± 143.06 ^aB^*	2764 ± 64.67 ^aB^*	5020 ± 125.51 ^aB^*	1591 ± 105.19 ^aB^*	2255 ± 92.40 ^aB^
120-RHMT-3	85.07 ± 0.51 ^bB^*	4494 ± 70.68 ^aB^*	2764 ± 72.14 ^aB^*	5192 ± 133.45 ^aB^*	1730 ± 139.20 ^aB^*	2427 ± 200.59 ^aA^
120-RHMT-5	85.35 ± 0.44 ^bB^*	5119 ± 94.55 ^bA^	3294 ± 63.07 ^bA^*	5587 ± 44.00 ^bA^*	1826 ± 35.68 ^aA^*	2293 ± 63.22 ^aA^
120-RHMT-7	85.62 ± 0.03 ^bB^*	3552 ± 9.81 ^cC^*	2466 ± 147.46 ^cB^	3744 ± 142.84 ^cC^*	1197 ± 160.46 ^bB^*	1278 ± 92.38 ^bC^*

Note: Values are means ± SD (*n* = 3). Different lowercase letters indicate significant differences among cycles within the same temperature, and different uppercase letters indicate significant differences among temperatures within the same cycle (EMMEANS, Bonferroni, *p* < 0.05). * indicates significant difference vs. NS (Dunnett, *p* < 0.05). Groups sharing at least one letter are not significantly different. NS was not included in the Temperature × Cycle two-way ANOVA.

**Table 2:** Two-way ANOVA results for the effects of treatment temperature, cycle number, and their interaction on the evaluated parameters of RHMT-modified starch.

Effect	Temperature	Cycle	Temperature × Cycle
*df* (*df*_1_, *df*_2_)	(2, 24)	(3, 24)	(6, 24)
PT (*F*, *p*)	181.28, *p* < 0.001	107.03, *p* < 0.001	4.77, *p* = 0.002
PV (*F*, *p*)	115.69, *p* < 0.001	18.23, *p* < 0.001	26.87, *p* < 0.001
TV (*F*, *p*)	13.06, *p* <0.001	38.54, *p* < 0.001	20.54, *p* < 0.001
FV (*F*, *p*)	159.56, *p* < 0.001	50.84, *p* < 0.001	63.60, *p* < 0.001
BD (*F*, *p*)	99.83, *p* < 0.001	17.59, *p* < 0.001	9.39, *p* < 0.001
SB (*F*, *p*)	105.20, *p* < 0.001	40.82, *p* < 0.001	13.51, *p* < 0.001
*K* (*F*, *p*)	51.48, *p* < 0.001	549.56, *p* < 0.001	138.41, *p* < 0.001
*n* (*F*, *p*)	0.57, *p* = 0.576	24.82, *p* < 0.001	13.29, *p* < 0.001
*k*_1_ (*F*, *p*)	14,018.03, *p* < 0.001	2961.62, *p* < 0.001	13,905.58, *p* < 0.001
*C*_1∞_ (*F*, *p*)	26,867.71, *p* < 0.001	165,127.88, *p* < 0.001	72,796.23, *p* < 0.001
*k*_2_ (*F*, *p*)	0.86, *p* = 0.436	6.83, *p* = 0.002	2.81, *p* = 0.032
*C_2∞_* (*F*, *p*)	394,950.99, *p* < 0.001	406,971.25, *p* < 0.001	117,606.38, *p* < 0.001
*J*_0*C*_ (*F*, p)	4.39, *p* = 0.024	0.07, *p* = 0.974	0.79, *p* = 0.590
*J*_1*C*_ (*F*, p)	1.11, *p* = 0.345	0.65, *p* = 0.591	0.44, *p* = 0.843
*λ*_1*C*_ (*F*, p)	3.59, *p* = 0.043	0.63, *p* = 0.601	1.59, *p* = 0.191
*μ*_0_ (*F*, p)	17.46, *p* < 0.001	0.18, *p* = 0.910	2.18, *p* = 0.081
*To* (*F*, p)	4.34, *p* = 0.025	0.43, *p* = 0.731	0.20, *p* = 0.975
*Tp* (*F*, p)	6.09, *p* = 0.007	0.28, *p* = 0.839	0.34, *p* = 0.910
*Te* (*F*, p)	5.72, *p* = 0.009	0.31, *p* = 0.822	0.49, *p* = 0.811
Δ*H* (*F*, p)	1.29, *p* = 0.294	0.34, *p* = 0.795	0.91, *p* = 0.505
*RC* (*F*, p)	14.64, *p* < 0.001	28.43, *p* < 0.001	30.39, *p* < 0.001
Aggregate size (*F*, p)	2.83, *p* = 0.079	0.18, *p* = 0.907	0.79, *p* = 0.582

Note: Two-way ANOVA was performed with temperature (*T*; three levels) and number of cycles (*C*; four levels) as fixed factors (*n* = 3 per *T* × *C* combination). The native starch (NS) control was excluded. Degrees of freedom are shown as (*df*_1_, *df*_2_). PT: pasting temperature; PV: peak viscosity; TV: trough viscosity; FV: final viscosity; BD: breakdown; SB: setback; *K*: consistency coefficient; *n*: flow behavior index; *k*_1_, *k*_2_: digestion rate constants; *C*_1*∞*_, *C*_2*∞*_: maximum digestion extents; *J*_0*C*_: instantaneous compliance; *J*_1*C*_: delayed elastic compliance; *λ*_1*C*_: retardation time; *μ*_0_: Newtonian viscosity; *To*, onset temperature; *Tp*, peak temperature; *Te*, conclusion gelatinization temperature; Δ*H*, gelatinization enthalpy; *RC*: relative crystallinity.

**Table 3:** Rheological parameters and in vitro digestion kinetic parameters of native starch and starch modified by repeated heat–moisture treatment at varying temperatures.

Samples	*K*	*n*	*R* ^2^	*k*_1_	*C*_1_ *_∞_*	*k*_2_	*C*_2_ *_∞_*
NS	321.942 ± 1.751	0.1758 ± 0.0131	0.963 ± 0.024	0.0998 ± 0.0001	38.3814 ± 0.0379	0.0133 ± 0.0001	24.5975 ± 0.0246
80-RHMT-1	241.866 ± 5.490 ^aA^*	0.2137 ± 0.0114 ^aA^*	0.958 ± 0.026	0.0374 ± 0.0001 ^aA^*	41.9200 ± 0.0419 ^aA^*	0.0161 ± 0.0002 ^aA^	29.6774 ± 0.1042 ^bA^*
80-RHMT-3	212.567 ± 1.220 ^bB^*	0.2854 ± 0.0317 ^bAB^*	0.981 ± 0.011	0.0641 ± 0.0006 ^bA^*	47.1990 ± 0.0472 ^bB^*	0.0221 ± 0.0002 ^abAB^	30.8485 ± 0.0308 ^aC^*
80-RHMT-5	171.215 ± 1.350 ^cC^*	0.3147 ± 0.0240 ^bcA^*	0.970 ± 0.004	0.0573 ± 0.0006 ^cA^*	51.6047 ± 0.0516 ^cB^*	0.0249 ± 0.0002 ^abA^*	29.3734 ± 0.0294 ^cB^*
80-RHMT-7	170.389 ± 2.287 ^cB^*	0.3426 ± 0.0069 ^cA^*	0.978 ± 0.003	0.0489 ± 0.0011 ^cA^*	66.9947 ± 0.0672^dB^*	0.0295 ± 0.0042 ^bA^*	19.001 ± 0.0187 ^dC^*
100-RHMT-1	209.026 ± 1.711 ^aB^*	0.2794 ± 0.0132 ^abB^*	0.976 ± 0.002	0.0501 ± 0.0001 ^aB^*	58.9500 ± 0.0600 ^cB^*	0.0202 ± 0.0001 ^aA^*	26.7767 ± 0.0252 ^bB^*
100-RHMT-3	191.733 ± 2.612 ^bC^*	0.3126 ± 0.0202 ^aA^*	0.979 ± 0.015	0.0500 ± 0.0001 ^bB^*	52.2533 ± 0.0558 ^aC^*	0.0300 ± 0.0002 ^aA^*	34.0733 ± 0.0351 ^aB^*
100-RHMT-5	189.135 ± 6.135 ^bB^*	0.3081 ± 0.0010 ^aA^*	0.983 ± 0.006	0.0202 ± 0.0002 ^bB^*	60.1200 ± 0.0600^dC^*	0.0201 ± 0.0001 ^aA^	24.7233 ± 0.0252 ^cC^*
100-RHMT-7	194.789 ± 3.906 ^bA^*	0.2582 ± 0.0131 ^bB^*	0.974 ± 0.008	0.0403 ± 0.0001 ^bB^*	57.8800 ± 0.0601 ^bA^*	0.0201 ± 0.0001 ^aA^*	20.9701 ± 0.0210 ^dB^*
120-RHMT-1	246.138 ± 5.300 ^aA^*	0.2559 ± 0.0142 ^aB^*	0.981 ± 0.011	0.0501 ± 0.0001 ^aC^*	63.3601 ± 0.0101 ^cC^*	0.0201 ± 0.0001 ^aA^	26.4367 ± 0.0306 ^dC^*
120-RHMT-3	240.012 ± 2.053 ^aA^*	0.2630 ± 0.0029 ^abB^*	0.984 ± 0.008	0.0402 ± 0.0001 ^aC^*	31.2602 ± 0.0173 ^aA^*	0.0201 ± 0.0001 ^aB^	61.5901 ± 0.0001 ^aA^*
120-RHMT-5	206.935 ± 1.075 ^bA^*	0.3004 ± 0.0065 ^bcA^*	0.986 ± 0.005	0.0501 ± 0.0001 ^bC^*	48.6721 ± 0.1001 ^bA^*	0.0201 ± 0.0001 ^abA^	43.3001 ± 0.0090 ^bA^*
120-RHMT-7	146.473 ± 3.458 ^cC^*	0.3098 ± 0.0039 ^cA^*	0.979 ± 0.002	0.0502 ± 0.0001 ^cC^*	67.1833 ± 0.1054 ^dC^*	0.0300 ± 0.0000 ^bA^*	26.5333 ± 0.0451 ^cA^

Note: Values are means ± SD (*n* = 3). Different lowercase letters indicate significant differences among cycles within the same temperature, and different uppercase letters indicate significant differences among temperatures within the same cycle (EMMEANS, Bonferroni, *p* < 0.05). * indicates significant difference vs. NS (Dunnett, *p* < 0.05). Groups sharing at least one letter are not significantly different. NS was not included in the Temperature × Cycle two-way ANOVA. *R*^2^ values are reported for descriptive purposes only and were not subjected to significance testing.

**Table 4:** Rheological characteristics of native starch and starch modified by repeated heat–moisture treatment at varying temperatures.

Samples	*J*_0*C*_ × 10^4^ (Pa^−1^)	*J*_1*C*_ × 10^4^ (Pa^−1^)	*λ*_1*C*_ (s)	*μ*_0_ × 10^−5^ (Pa·s)	*R* ^2^
NS	1.5345 ± 0.0217	9.4878 ± 0.3193	11.2281 ± 0.1210	5.5827 ± 0.0607	0.9340 ± 0.0030
80-RHMT-1	1.2574 ± 0.0180 ^aB^*	4.1979 ± 0.0713 ^aA^*	15.8705 ± 0.1871 ^aB^*	4.9226 ± 0.0461 ^aB^*	0.9606 ± 0.0025
80-RHMT-3	1.4579 ± 0.0254 ^aB^*	5.3219 ± 0.0809 ^aA^*	16.3686 ± 0.1527 ^aB^*	3.3507 ± 0.0519 ^aB^*	0.9041 ± 0.0030
80-RHMT-5	1.5962 ± 0.0233 ^aB^*	5.1812 ± 0.0671 ^aA^*	17.6129 ± 0.1457 ^aB^*	2.2257 ± 0.0461 ^aB^*	0.9461 ± 0.0030
80-RHMT-7	1.7081 ± 0.0236 ^aB^*	7.6411 ± 0.0791 ^aA^*	58.0744 ± 0.4380 ^aB^*	2.1862 ± 0.0486 ^aB^*	0.9806 ± 0.0025
100-RHMT-1	1.1209 ± 0.0181 ^aAB^*	4.3582 ± 0.0616 ^aA^*	14.3079 ± 0.1452 ^aA^*	3.2573 ± 0.0553 ^aC^*	0.9726 ± 0.0025
100-RHMT-3	1.3146 ± 0.0206 ^aAB^*	6.644 ± 0.0716 ^aA^*	55.7624 ± 0.4383 ^aA^*	4.6632 ± 0.0557 ^aC^*	0.9801 ± 0.0020
100-RHMT-5	1.6031 ± 0.0218 ^aAB^*	7.3762 ± 0.0718 ^aA^*	66.8322 ± 0.4583 ^aA^*	4.8407 ± 0.0547 ^aC^*	0.9726 ± 0.0025
100-RHMT-7	1.6026 ± 0.0197 ^aAB^*	5.0714 ± 0.0579 ^aA^*	54.9237 ± 0.4378 ^aA^*	7.7974 ± 0.0723 ^aC^*	0.9351 ± 0.0030
120-RHMT-1	1.7158 ± 0.0228 ^aA^*	3.8284 ± 0.0563 ^aA^*	9.7546 ± 0.1421 ^aA^*	6.6341 ± 0.0608 ^aA^*	0.9060 ± 0.0129
120-RHMT-3	1.6729 ± 0.0217 ^aA^*	3.7034 ± 0.0507 ^aA^*	13.8920 ± 0.1779 ^aA^*	9.2924 ± 0.0759 ^aA^*	0.9006 ± 0.0015
120-RHMT-5	1.5573 ± 0.0227 ^aA^	4.8154 ± 0.0532 ^aA^*	17.2171 ± 0.1572 ^aA^*	4.6745 ± 0.0546 ^aA^*	0.9606 ± 0.0025
120-RHMT-7	0.8500 ± 0.0174 ^aA^*	4.8854 ± 0.0534 ^aA^*	35.6528 ± 0.4323 ^aA^*	8.5000 ± 0.0674 ^aA^*	0.9690 ± 0.0020

Note: Values are expressed as means ± SD (*n* = 3). Different lowercase letters indicate significant differences among cycles within the same temperature, and different uppercase letters indicate significant differences among temperatures within the same cycle (EMMEANS, Bonferroni, *p* < 0.05). * indicates a significant difference compared to the native starch (NS) group (Dunnett’s test, *p* < 0.05). NS was excluded from the two-way ANOVA. *R*^2^ values are reported for descriptive purposes only and were not subjected to significance testing. *J*_0*C*_: instantaneous compliance; *J*_1*C*_: delayed elastic compliance; *λ*_1*C*_: retardation time; *μ*_0_: Newtonian viscosity.

**Table 5:** Thermal and structural characteristics of native starch and starch modified by repeated heat–moisture treatment at varying temperatures.

Samples	*To* (°C)	*Tp* (°C)	*Te* (°C)	Δ*H* (J/g)	*RC* (%)	Aggregate Size (μm)
NS	68.07 ± 0.15	74.17 ± 0.06	79.70 ± 0.53	11.51 ± 0.36	14.39 ± 0.17	17.71 ± 0.32
80-RHMT-1	72.57 ± 0.12 ^aB^*	78.50 ± 0.26 ^aB^*	82.70 ± 0.26 ^aB^*	10.97 ± 0.12 ^aA^*	14.31 ± 0.71 ^bcC^	25.86 ± 0.55 ^aB^*
80-RHMT-3	76.83 ± 0.15 ^aB^*	80.77 ± 0.06 ^aB^*	84.53 ± 0.21 ^aB^*	9.68 ± 0.27 ^aA^*	15.83 ± 0.53 ^aA^*	21.34 ± 0.41 ^aB^*
80-RHMT-5	77.76 ± 0.15 ^aB^*	81.73 ± 0.15 ^aB^*	85.50 ± 0.26 ^aB^*	9.75 ± 0.14 ^aA^*	14.25 ± 0.68 ^bB^	24.25 ± 0.47 ^aB^*
80-RHMT-7	78.93 ± 0.16 ^aB^*	82.13 ± 0.14 ^aB^*	85.77 ± 0.21 ^aB^*	9.82 ± 0.10 ^aA^*	13.04 ± 0.36 ^cA^*	30.03 ± 0.58 ^aAB^*
100-RHMT-1	73.70 ± 0.62 ^aAB^*	81.73 ± 0.21 ^aA^*	86.07 ± 0.15 ^aAB^*	9.15 ± 0.08 ^aA^*	17.72 ± 0.22 ^aA^*	31.38 ± 0.60 ^aAB^*
100-RHMT-3	78.33 ± 0.12 ^aAB^*	83.00 ± 0.10 ^aA^*	87.30 ± 0.17 ^aAB^*	9.23 ± 0.07 ^aA^*	14.37 ± 0.50 ^bcB^	25.64 ± 0.51 ^aAB^*
100-RHMT-5	79.53 ± 0.06 ^aAB^*	83.63 ± 0.12 ^aA^*	87.70 ± 0.10 ^aAB^*	8.96 ± 0.16 ^aA^*	14.99 ± 0.35 ^bAB^	30.50 ± 0.57 ^aAB^*
100-RHMT-7	80.20 ± 0.20 ^aAB^*	84.50 ± 0.10 ^aA^*	88.43 ± 0.21 ^aAB^*	7.40 ± 0.16 ^aA^*	13.57 ± 0.78 ^cA^	32.59 ± 0.62 ^aAB^*
120-RHMT-1	76.67 ± 0.21 ^aA^*	82.73 ± 0.23 ^aA^*	86.83 ± 0.25 ^aA^*	8.92 ± 0.12 ^aA^*	15.42 ± 0.43 ^aB^	28.30 ± 0.52 ^aA^*
120-RHMT-3	79.23 ± 0.14 ^aA^*	83.60 ± 0.00 ^aA^*	87.80 ± 0.00 ^aA^*	8.24 ± 0.06 ^aA^*	13.78 ± 0.60 ^cC^*	28.05 ± 0.52 ^aA^*
120-RHMT-5	79.53 ± 0.15 ^aA^*	84.50 ± 0.10 ^aA^*	88.90 ± 0.36 ^aA^*	6.65 ± 0.09 ^aA^*	15.60 ± 0.28 ^aA^*	34.16 ± 0.63 ^aA^*
120-RHMT-7	80.83 ± 0.06 ^aA^*	85.57 ± 0.15 ^aA^*	90.47 ± 0.25 ^aA^*	3.25 ± 0.06 ^aA^*	14.00 ± 0.37 ^bA^	36.20 ± 0.67 ^aA^*

Note: Values are means ± SD (*n* = 3). Different lowercase letters indicate significant differences among cycles within the same temperature, and different uppercase letters indicate significant differences among temperatures within the same cycle (EMMEANS, Bonferroni, *p* < 0.05). * indicates significant difference vs. NS (Dunnett, *p* < 0.05). Groups sharing at least one letter are not significantly different. NS was excluded from the two-way ANOVA. *To*, onset gelatinization temperature; *Tp*, peak gelatinization temperature; *Te*, conclusion gelatinization temperature; Δ*H*, gelatinization enthalpy; *RC*, relative crystallinity.

## Data Availability

All original contributions from this study are included in the article/Supplementary Material. Further inquiries can be directed to the corresponding author.

## Supplementary Materials

The following supporting information can be downloaded at: https://www.mdpi.com/article/10.3390/foods15132308/s1, Table S1: Impact of RHMT temperature on the in vitro digestibility and short-range order of hard proso millet starch. Table S2: Two-way ANOVA results for the effects of temperature (*T*), number of cycles (*C*), and their interaction (*T* × *C*) on digestive and structural characteristics of native and RHMT-modified starch.

## Abbreviations

The following abbreviations are used in this manuscript:

HMT	Heat–moisture treatment
RHMT	Repeated heat–moisture treatment
NS	Native starch
PT	Pasting temperature
PV	Peak viscosity
TV	Trough viscosity
FV	Final viscosity
SB	Setback viscosity
BD	Breakdown viscosity
LOS	Logarithm of slope
CPS	Combined parallel-sequential
RDS	Rapidly digestible starch
SDS	Slowly digestible starch
RS	Resistant starch
RC	Relative crystallinity
