# Supplementary material for: Effect of Repeated Heat–Moisture Treatment Temperature on the Multi-Scale Structure, Physicochemical Properties, Rheological Behavior, and In Vitro Digestibility of Hard Proso Millet Starch
Source: Foods. 2026 Jun 29;15(13):2308. doi: 10.3390/foods15132308 (PMC13362534; doi:10.3390/foods15132308)
Supplement: Supplementary file 1 [file foods-15-02308-s001.zip › foods-4316617-supplementary.pdf]

Table captions:

Table S1. Impact of RHMT temperature on the in vitro digestibility and short-range order of hard proso millet starch.

Table S2. Two-way ANOVA results for the effects of temperature (*T*), number of cycles (*C*), and their interaction ($T \times C$) on digestive and structural characteristics of native and RHMT-modified starch.

Table S1

Impact of RHMT temperature on the in vitro digestibility and short-range order of hard proso millet starch.

Samples	RDS(%)	SDS(%)	RS(%)	1047 cm^{-1}/ 1022 cm^{-1}	995 cm^{-1}/ 1022 cm^{-1}
**NS**	12.52 ± 0.41	33.70 ± 1.34	53.78 ± 1.02	0.918 ± 0.008	0.886 ± 0.007
**80-RHMT-1**	17.00 ± 0.64^{aC*}	42.55 ± 2.53^{aAB*}	40.45 ± 2.77^{aA*}	0.902 ± 0.007^{aA}	0.869 ± 0.006^{aAB}
**80-RHMT-3**	33.94 ± 1.89^{bC*}	41.66 ± 1.20^{abB*}	24.40 ±2.92^{bA*}	0.908 ± 0.006^{aA}	0.875 ± 0.008^{aA}
**80-RHMT-5**	36.90 ± 0.60^{cB*}	40.24 ± 0.80^{bB*}	22.87 ± 0.23^{bA*}	0.889 ± 0.007^{abA*}	0.857 ± 0.007^{abA*}
**80-RHMT-7**	40.51 ± 1.20^{dB*}	40.20 ± 0.33^{bB*}	19.29 ± 1.11^{cB*}	0.873 ± 0.008^{bA*}	0.841 ± 0.006^{bA*}
**100-RHMT-1**	32.89 ± 1.24^{bB*}	40.79 ± 0.69^{cB*}	26.32 ± 1.01^{aB*}	0.913 ± 0.006^{aA}	0.881 ± 0.008^{aA}
**100-RHMT-3**	36.09 ± 0.91^{cB*}	40.15 ± 0.69^{cB*}	23.76 ± 0.43^{abA*}	0.882 ± 0.007^{bB*}	0.850 ± 0.007^{bB*}
**100-RHMT-5**	29.96 ± 0.20^{aC*}	47.44 ± 0.68^{aA*}	22.60 ± 0.55^{bA*}	0.891 ± 0.008^{bA*}	0.858 ± 0.006^{bA*}
**100-RHMT-7**	30.50 ± 1.37^{aC*}	43.74 ± 0.73^{bA*}	25.75 ± 1.86^{abA*}	0.876 ± 0.007^{bA*}	0.843 ± 0.008^{bA*}
**120-RHMT-1**	36.02 ± 1.68^{aA*}	43.58 ± 3.31^{aA*}	20.40 ± 1.79^{aC*}	0.885 ± 0.008^{aB*}	0.852 ± 0.007^{aB*}
**120-RHMT-3**	38.59 ± 0.31^{bA*}	45.67 ± 0.49^{aA*}	15.74 ± 0.72^{bB*}	0.863 ± 0.007^{bC*}	0.830 ± 0.008^{aB*}
**120-RHMT-5**	47.66 ± 0.35^{cA*}	40.34 ± 0.79^{bB*}	12.00 ± 0.50^{cB*}	0.847 ± 0.009^{bB*}	0.813 ± 0.007^{bB*}
**120-RHMT-7**	58.73 ± 0.49^{dA*}	36.64 ± 0.21^{cC*}	4.63 ± 1.01^{dC*}	0.824 ± 0.006^{cB*}	0.789 ± 0.009^{cB*}

Note: Values are means ± SD (n = 3). Different lowercase letters indicate significant differences among cycles within the same temperature, and different uppercase letters indicate significant differences among temperatures within the same cycle (EMMEANS, Bonferroni, $p < 0.05$). * indicates significant difference vs. NS (Dunnett, $p < 0.05$). Groups sharing at least one letter are not significantly different. NS was not included in the Temperature × Cycle two-way ANOVA.

Table S2

Two-way ANOVA results for the effects of temperature (*T*), number of cycles (*C*), and their interaction (*T* × *C*) on digestive and structural characteristics of native and RHMT-modified starch.

**Effect**	*df* (df_1, df_2)	**RDS (*F*, *p*)**	**SDS (*F*, *p*)**	**RS (*F*, *p*)**	**1047 cm^{-1}/ 1022 cm^{-1} (*F*, *p*)**	**995 cm^{-1}/ 1022 cm^{-1} (*F*, *p*)**
**Temperature**	(2, 24)	764.38, $p < 0.001$	12.82, $p < 0.001$	303.51, $p < 0.001$	105.33, $p < 0.001$	74.36, $p < 0.001$
**Cycle**	(3, 24)	372.29, $p < 0.001$	13.29, $p < 0.001$	124.25, $p < 0.001$	53.77, $p < 0.001$	45.91, $p < 0.001$
**Temperature × Cycle**	(6, 24)	188.57, $p < 0.001$	39.58, $p < 0.001$	34.47, $p < 0.001$	6.66, $p < 0.001$	6.69, $p < 0.001$

Note: Two-way ANOVA was performed with temperature (*T*; three levels) and number of cycles (*C*; four levels) as fixed factors (n = 3 per T × C combination). The NS control was excluded. Degrees of freedom are shown as (df_1, df_2).
